# Versatile Coordination Modes of Multidentate Neutral Amine Ligands with Group 1 Metal Cations

**DOI:** 10.1021/acs.inorgchem.1c03786

**Published:** 2022-02-11

**Authors:** Nathan Davison, Ke Zhou, Paul G. Waddell, Corinne Wills, Casey Dixon, Shu-Xian Hu, Erli Lu

**Affiliations:** † Chemistry-School of Natural and Environmental Sciences, Newcastle University, Newcastle upon Tyne, United Kingdom, NE1 7RU; ‡ Beijing Computational Science Research Center, Beijing 100193, China; § College of Chemistry and Environmental Science & Shaanxi Key Laboratory of Catalysis & Institute of Theoretical and Computational Chemistry, Shaanxi University of Technology. Hanzhong 723000, Shaanxi Province, China

## Abstract

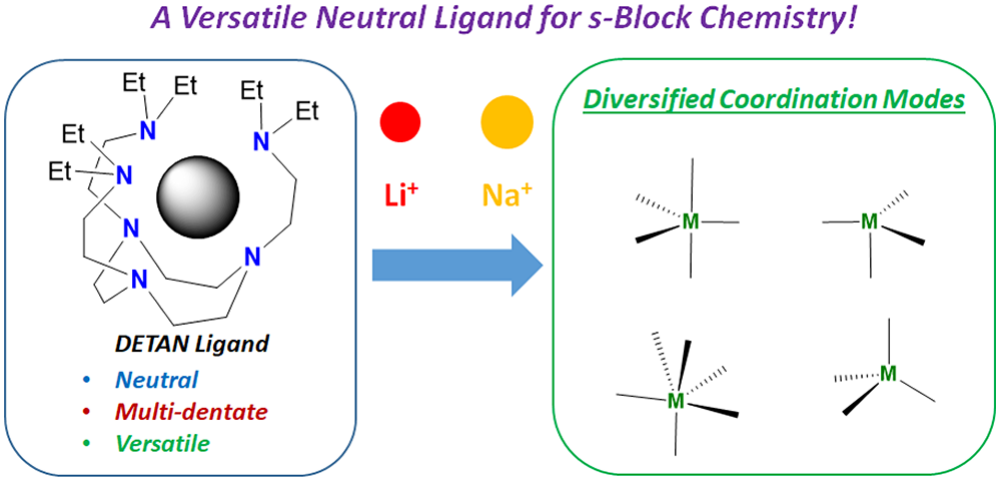

This work comprehensively investigated the coordination chemistry of a *hexa*-dentate neutral amine ligand, namely, *N,N′,N”*-*tris*-(2-*N*-diethylaminoethyl)-1,4,7-triaza-cyclononane (DETAN), with group-1 metal cations (Li^+^, Na^+^, K^+^, Rb^+^, Cs^+^). Versatile coordination modes were observed, from four-coordinate trigonal pyramidal to six-coordinate trigonal prismatic, depending on the metal ionic radii and metal’s substituent. For comparison, the coordination chemistry of a *tetra*-dentate *tris*-[2-(dimethylamino)ethyl]amine (Me_6_Tren) ligand was also studied. This work defines the available coordination modes of two multidentate amine ligands (DETAN and Me_6_Tren), guiding future applications of these ligands for pursuing highly reactive and elusive s-block and rare-earth metal complexes.

## Abstract 2

The coordination modes of a newly developed *hexa*-dentate neutral amine ligand (DETAN) with Group 1 metals were comprehensively studied in solid state. Depending on the metal’s ionic radii and its functional groups (E of the M−E bond), the DETAN ligand could adopt four-, five-, or six-coordinate geometries, demonstrating the ligand’s versatility and flexibility.

## Introduction

Ligand design is at the center stage of coordination chemistry and plays an essential role in catalysis. A well-designed ligand is a prerequisite for isolating and analyzing highly reactive and elusive metal coordination species. The knowledge, in return, enables chemists to design new catalytic reactions. Several recent breakthroughs in coordination chemistry highlighted the importance of ligand design, such as a tripodal *tris*-anionic amide ligand stabilized uranium terminal nitride,^1^ and *N*-heterocyclic carbene (NHC) or cyclo-amino alkyl carbene (cAAC)-stabilized low-valent boron^2,3^ and beryllium^4,5^ complexes.

As in the d-/f-/p-block metal chemistry, ligand design is equally crucial in s-block Group 1 metal chemistry. Highly reactive Group 1 metal species play essential roles in numerous catalytic processes and act as ubiquitous reagents, such as organolithium reagents,^6^ Lochman Schlosser superbases,^7^ and the plethora of Group 1 metal salts used as additives in organic synthesis.^8,9^ These systems are highly complicated, and they usually involve aggregating/clustering of multimetallic species. To understand their mechanisms, coordination chemists used several ligands to “trap” the highly reactive and elusive species and synthesize model complexes.

Therefore, designing new bespoke ligands and understanding their applicable range (such as metal ionic radii range) and corresponding coordination modes is essential to synthesizing such highly reactive and elusive metal complexes. However, designing bespoke ligands for Group 1 metals is more difficult than for the d-/f-/p-block and Group 2 metals. The difficulties are caused by two factors. (1) From a charge balance perspective, the monovalent Group 1 metal cation rules out the usage of anionic building blocks for heteroleptic complexes. For example, the massively successful anionic cyclopentadienyl (Cp) and *beta*-diketiminate (BDI) families are of little use in Group 1 chemistry. (2) The Group 1 metal cations feature an ns^0^ valence shell electronic structure, and form ionic bonds with ligand atoms: the metal-to-ligand backdonation is very weak, if there is any. Therefore, they cannot take advantage of donor–acceptor building blocks, such as the popular *N*-heterocyclic carbenes (NHCs)^10^ and cAACs.^11^

Multidentate neutral amine ligands are arguably the most successful ligand family in Group 1 metal chemistry.^12−16^ Their two key advantages are (1) synthetic availability and tuneability; and (2) chemical robustness (the C–N bond is more stable than the C–O bond). The denticity of the multidentate amine ligands plays an essential role in their coordination chemistry. The most-used ligands are *bis*- and *tris*-dentate, such as *tetra*-methyl ethylenediamine (TMEDA),^17^ (−)-sparteine,^18^ (*R*,*R*)-*N*,*N*,*N*′,*N*′-tetramethyl-1,2-diaminocyclohexane [(*R*,*R*)-TMCDA]^19^ and *N*,*N*,*N*′,*N*″,*N*′′-pentamethyldiethylenetriamine (PMDTA).^20,21^ The *bis*- and *tris*-dentate amine ligands (L) succeeded in isolating monomers of sterically bulky lithium alkyl complexes [LiR(L)].^17−20^*Tetra*-dentate amine ligands, such as *tris*-[2-(dimethylamino)ethyl]amine (Me_6_Tren), also has been used in Group 1 chemistry.^22−31^ Efforts were also made to combine multidentate neutral amine donors and anionic donors, such as a cyclen-derived *tetra*-dentate (NNNN) macrocylic anionic ligand developed by Okuda and co-workers.^32^ However, the *bis*-, *tris*- and *tetra*-dentate ligands could not provide sufficient kinetic protection for isolating more reactive species.^17^ Higher-dentate amine ligands are necessary. For example, a *nona*-dentate per-aza cryptand[2,2,2] was designed by Dye and co-workers to isolate a thermally stable organic electride.^33^ Recently, we designed a *hexa*-dentate ligand, namely, *N*,*N*′,*N*′′-*tris*-(2-*N*-diethylaminoethyl)-1,4,7-triaza-cyclononane (DETAN), and isolated the first methyllithium (MeLi) monomer.^34^

Our DETAN ligand combines a semirigid 1,4,7-triazacyclononane (TACN) macrocyclic backbone and three flexible coordinative side arms, which feature good thermodynamic robustness (no fragile C–O bond). Compared to Dye’s per-aza cryptand[2,2,2],^33^ the DETAN is more flexible and could accommodate reactive M–E metal functional groups with variable sizes, such as metal terminal imides/phosphinidenes (M=NR/M=PR) and nitrides/phosphides (M≡N/M≡P). Moreover, unlike the rigid per-aza cryptand[2,2,2], DETAN’s flexible side arms could decoordinate, allowing Lewis basic organic/small molecular substrates to approach the metal center and facilitate subsequent reactivity studies.

The excellent kinetic protection and thermodynamic robustness, as well as the flexible side arms, make DETAN an attractive ligand for pursuing long sought-after and highly reactive metal complexes, such as monomeric Group 1 metal amide/hydride,^35^ Group 2 metal terminal imides,^36^ and divalent rare-earth metal terminal imides.^37−39^ A prerequisite for these applications is a comprehensive understanding of the DETAN’s coordination modes. In this work, employing Group 1 metal halides and tetraphenylborates as a platform, we mapped out the diversified coordination modes of the DETAN ligand and how it changes in accordance with the metal ionic radii and the M–E functional groups. For comparison, the *tetra*-dentate Me_6_Tren ligand was studied as well. The results are reported below.

## Synthesis and Characterization

**Scheme 1:**
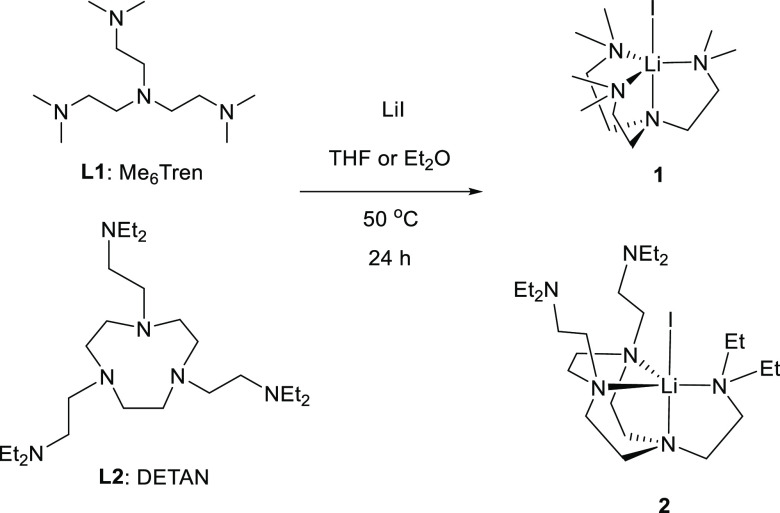
Reactions To Produce [Li(I)(κ^*4*^-*N*,*N*′,*N*″,*N*′′′-Me_6_Tren)] (**1**) and [Li(I)(κ^4^-*N*,*N*′,*N*″,*N*′′′-DETAN)] (**2**)

We first treated lithium iodide (LiI) with Me_6_Tren and DETAN in *d*^8^-THF, respectively. The NMR scale reactions were monitored by ^1^H and ^7^Li NMR spectra, indicating full conversions within 24 h at room temperature. The reactions were subsequently scaled up, employing diethyl ether (Et_2_O) or THF as the solvent, to yield complexes **1** and **2** in 96% (**1**) and 79% (**2**) yields, respectively (Scheme 1). Complexes [Li(I)(κ^4^-*N*,*N*′,*N*″,*N*′′′-Me_6_Tren)] (**1**) and [Li(I)(κ^4^-*N*,*N*′,*N*″,*N*′′′-DETAN)] (**2**) were obtained as white crystalline solids. It is worth mentioning that complex **2** is more soluble than complex **1** in most organic solvents. Specifically, complex **1** is soluble in THF, sparingly soluble in benzene/toluene, and insoluble in Et_2_O and hexane. In comparison, complex **2** is soluble in THF/Et_2_O/toluene/benzene, and insoluble in hexane. We attribute the better solubility of **2** to the side arms of the DETAN ligand. Complexes **1** and **2** are stable at room temperature indefinitely, and they are extremely hygroscopic when exposed to air, to produce intractable mixtures containing free DETAN/Me_6_Tren ligands.

**Figure 1:**
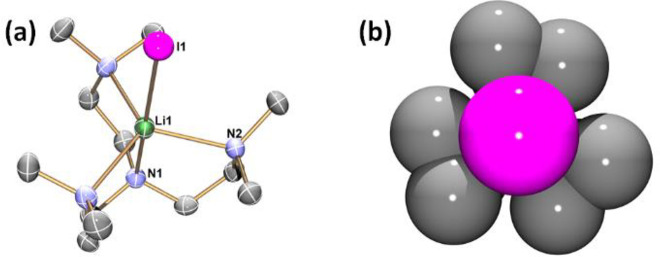
(a) X-ray crystal structure of [Li(I)(κ^4^-*N*,*N*′,*N*″,*N*′′′-Me_6_Tren)] (**1**) at 150 K with 50% probability ellipsoids. Hydrogen atoms are omitted for clarity. Only atoms of the crystallographically independent fragment are labeled. (b) Top-view space-filling presentation for **1** against the Li1–I1 bond. The selected bond distances are Li1–I1, 2.98(2) Å; Li1–N1, 2.19(3) Å; Li1–N2 2.221(6) Å. The selected bond angles are I1–Li1–N1, 180.0°; I1–Li1–N2, 98.4(5)°; N2–Li1–N1, 81.6(5)°; N2–Li1–N2, 117.9(2)°. The atomic color codes: Li (forest green); C (gray); N (blue); and I (magenta).

**Figure 2:**
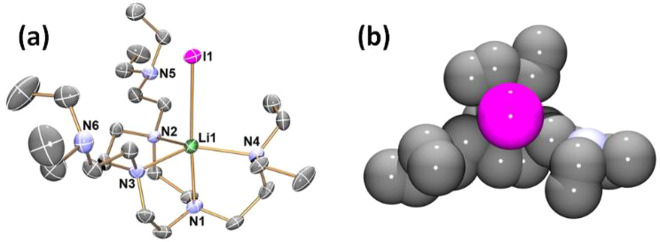
(a) X-ray crystal structure of [Li(I)(κ^4^-*N*,*N*′,*N*″,*N*′′′-DETAN)] (**2**) at 150 K with 50% probability ellipsoids. Hydrogen atoms are omitted for the sake of clarity. (b) Top-view space-filling presentation for **2** against the Li1–I1 bond. The selected bond distances (Å) and angles (deg) are Li1–I1, 3.110(5); Li1–N1, 2.173(6); Li1–N2 2.149(6); Li1–N3 2.180(6); Li1–N4 2.179(6); I1–Li1–N1, 175.6(2); N2–Li1–N3, 86.3(2); N3–Li1–N4 131.2(3); N4–Li1–N2 137.2(3); N1–Li1–N3, 81.1(2); N1–Li1–N4, 82.7(2); N1–Li1–N2 84.4(2); N4–Li1–I1, 93.40(19); N2–Li1–I1, 99.9(2); N3–Li1–I1, 100.1(2). The atomic color codes: Li (forest green); C (gray); N (blue); I (magenta).

**Chart 1:**
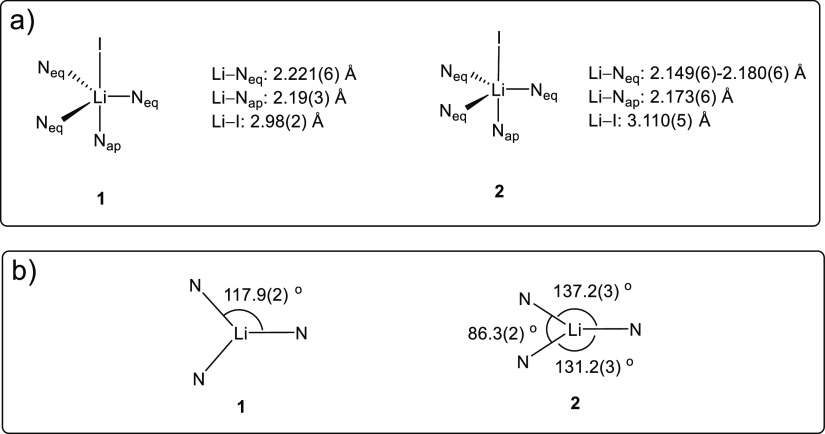
(a) Schematic Representations of the Structures of **1** and **2**; (b) The Equatorial Angle Distributions (Apical View) of **1** and **2**

Single crystals of complexes **1** and **2** suitable for SCXRD studies were obtained from THF (**1**) and Et_2_O (**2**) solutions at room temperature (**1**) or −35 °C (**2**). Their structures are exhibited in Figures 1 and 2, respectively. Both **1** and **2** feature a trigonal bipyramidal coordination geometry surrounding the five-coordinate Li^+^ center. Three equatorial and one apical positions are occupied by neutral N donors, while the other apical position is occupied by an anionic iodide (I^–^) donor. In complex **1**, all four N donors of the Me_6_Tren ligand coordinate to the Li^+^ center. In comparison, in complex **2**, only four out of the six N donors coordinate to the Li^+^ center. A closer examination of the structures of **1** and **2** reveals that, although both are trigonal bipyramidal, their Li–I bond lengths are significantly different (Chart 1). The Li–I bond in **1** (2.98(2) Å) is significantly shorter than the one in **2** (3.110(5) Å). The Li–I bonds in **1** and **2** are among the longest ones of their kind. They are longer than most reported terminal, nonbridging Li–I bonds (2.67–2.87 Å) by over 0.1 Å.^40−44^ Only one example of a longer terminal Li–I bond (3.233(14) Å) was reported, but the data was low quality (*R*_int_ = 10.94%; *d*_min_ = 45.8° 2θ).^45^ The Li–I bond lengths in **1** (2.98(2) Å) sits at the boundary of the sum of the ionic radii of Li^+^ (0.9 Å; coordination number (CN) = 6) and I^–^ (2.06 Å),^46^ and the Li–I bond length in **2** (3.110(5) Å) is ca. 0.15 Å longer than the sum of their ionic radii. Given the similar level of steric congestion around the Li–I unit in **1** and **2** (as demonstrated by the space-filling models in Figures 1b and 2b), the difference in the Li–I bond length is intriguing.

Comparing schematic structural representations of **1** and **2** could help readers understand their structural differences (Chart 1). It is noticeable that the Li–N bonds in **2** are shorter than those in **1** (Chart 1a), and the equatorial angle distributions are different (Chart 1b), although the sum of equatorial angles (∑∠_eq_) are both close to 360° (353.7° for **1**, and 354.8° for **2**).

We conducted density functional theory (DFT) calculations to optimize the structures of complexes **1** and **2**, employing four types of functionals (PBE, B3LYP, B3PW91, and PBE0).^47−49^ The relativistic effective core potential basis set SDD was used for the iodine,^50^ while def-TZVPP or 6-31*G*/6-31+G^50,51^ were used for other nuclei. Dispersion forces, weak intermolecular interactions, and THF solvent effects were also considered (see the Supporting Information). All the calculation methods reproduced the experimental structures with good accuracies, including the differences in the Li–I and Li–N bonds between **1** and **2**. The NPA charge calculations and the NLMO calculations indicate that the Li–I bonds in complexes **1** and **2**, although they differ by ca. 0.13 Å in bond lengths, both are predominantly ionic and have similar electrostatic environments. However, the energy decomposition analysis (EDA) of the Li–I bonds reveal that, in complex **2**, the Li–I bond is slightly more covalent than that in **1** (see Tables S1–S4 in the Supporting Information). Given the similarity of the underlying electronic structures between **1** and **2**, we attribute the difference in their Li–I bond lengths to slightly different steric environments.

**Chart 2:**
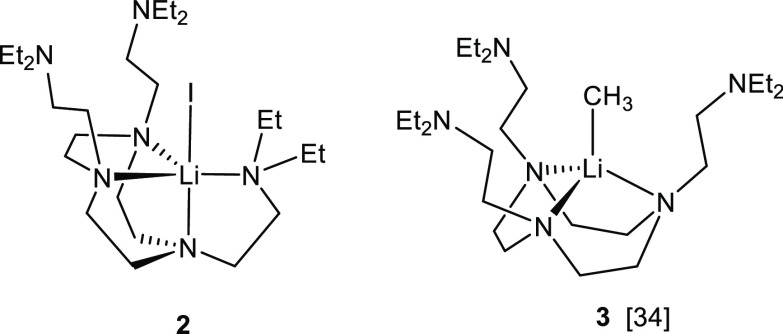
Comparison between Complexes **2** and **3**a

**Chart 3:**
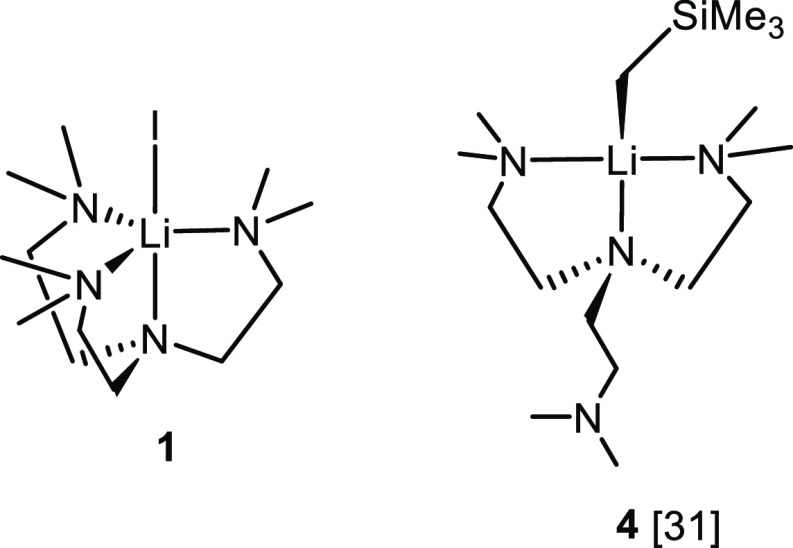
Comparison between Complexes **1** and **4**a

A comparison between the structures of complex **2** and our previous reported [Li(CH_3_)(κ^3^-*N*,*N*′,*N*′′-DETAN)] (**3**) is intriguing (see Chart 2).^34^ The DETAN ligand in **2** adopts a κ^4^-*N*,*N*′,*N*″,*N*′′′ mode, while in **3** it is in a κ^3^-*N*,*N*′,*N*′′mode. The key difference between **2** and **3** is that one out of the three side arms of the DETAN coordinates in **2**, while none of them coordinates in **3**. We attribute the difference to the increased steric congestion of **3**, compared to **2**. The methyl (−CH_3_) functional group is slightly bigger than the iodide (−I), and the Li–C bond is much shorter than the Li–I bond (2.099(5) Å vs 3.110(5) Å). Similar decoordinating of side arm is observed for the Me_6_Tren ligand as well. In the [Li(I)(κ^4^-*N*,*N*′,*N*″,*N*′′′-Me_6_Tren)] (**1**), all the three side arms coordinate to form a five-coordinate, trigonal bipyramidal geometry. In comparison, with the presence of a bulkier Li-CH_2_SiMe_3_ group, one of the side arms decoordinates to form a four-coordinate distorted trigonal pyramidal geometry in [Li(CH_2_SiMe_3_)(κ^3^-*N*,*N*′,*N*′′- Me_6_Tren)] (**4**) (Chart 3), where **4** was recently reported by us.^31^

a Data taken from ref (34). The DETAN ligand is in κ_4_-*N*,*N*′,*N*″,*N*′′′ (**2**) and κ_3_-*N*,*N*′,*N*′′ (**3**) mode, respectively.

a Data taken from ref (31). The Me_6_Tren ligand is in κ_4_-*N*,*N*′,*N*″,*N*′′′ (**1**) and κ_3_-*N*,*N*′,*N*′′ (**4**) mode, respectively.

**Scheme 2:**
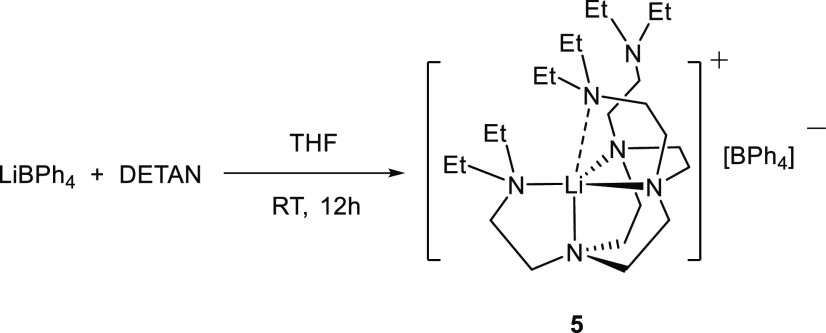
Reaction To Produce [Li(κ^4^-*N*,*N*′,*N*″,*N*′′′-DETAN)][BPh_4_] (**5**)

**Figure 3:**
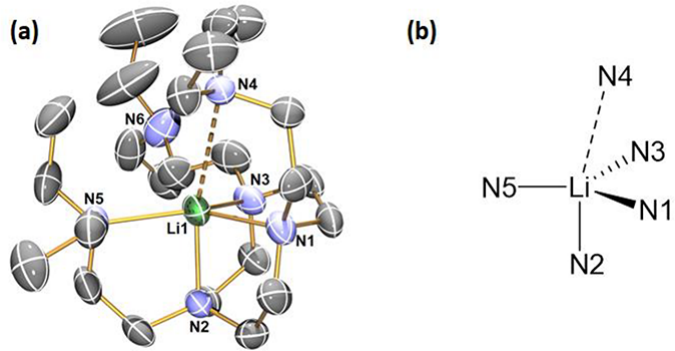
(a) X-ray crystal structure of [Li(κ^4^-*N*,*N*′,*N*″,*N*′′′-DETAN)][BPh_4_] (**5**) at 150 K with 50% probability ellipsoids. Hydrogen atoms, the minor disorder component and the [BPh_4_]^−^ anion are omitted for the sake of clarity. (b) Schematic representation of the coordination geometry of the Li^+^ center. The selected bond distances are as follows: Li1–N1, 2.104(4) Å; Li1–N2, 2.091(4) Å; Li1–N3, 2.135(4) Å; Li1–N5, 2.206(6) Å; and Li1**···**N5, 3.022 Å. The selected bond angles are as follows: N1–Li1–N3, 87.41(15)°; N3–Li1–N5, 131.7(2)°; and N5–Li1–N1, 138.5(2)°. The atomic color codes: Li (forest green), C (gray), N (blue).

In addition to lithium iodide, lithium tetraphenylborate (LiBPh_4_) also reacts with the DETAN ligand, to produce a separated ion pair (SIP) complex [Li(κ^4^-*N*,*N*′,*N*″,*N*′′′-DETAN)][BPh_4_] (**5**) (Scheme 2). Complex **5** was obtained in 84% yield as a white crystalline solid. Single crystals suitable for SCXRD study were obtained from a Et_2_O/THF mixed solution at −35 °C. The crystal structure of **5** is displayed in Figure 3. The coordination geometry of the Li^+^ center in **5** is best described as a trigonal pyramidal (Li1–N1–N3–N5-N2) capped by a weak N**···**Li interaction (N4**···**Li1) (Figure 3b). The N4**···**Li1 distance (3.022 Å) is significantly longer than other Li–N distances (2.09–2.21 Å) in the molecule, but shorter than the sum of the van der Waals radii for Li and N (3.36 Å^52^), which support that the N4**···**Li1 is a weak interaction, while the other Li–N interactions are dative bonds. The coordination geometry of the [Li(DETAN)]^+^ cationic fragment is similar to a Me_6_Tren-Li SIP complex [Li(Me_6_Tren)][AlH_2_(HMDS)_2_] reported by Mulvey and co-workers in 2018.^53^

**Scheme 3:**
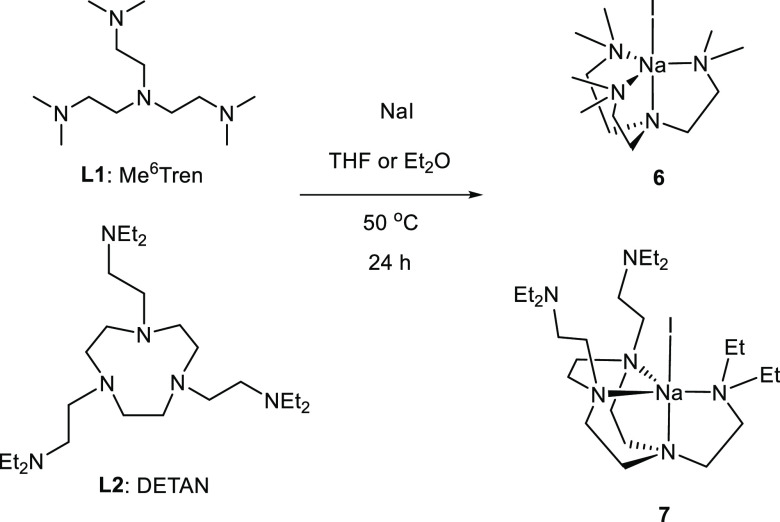
Reactions To Produce [Na(I)(κ^*4*^-*N*,*N*′,*N*″,*N*′′′-Me_6_Tren)] (**6**) and [Na(I)(DETAN)] (**7**)

Beyond lithium, we tested the reactions of the Me_6_Tren and DETAN ligands to other Group 1 metals. As the immediate neighbor of Li^+^, Na^+^ has a significantly larger ionic radius (*r*) compared to Li^+^ (coordination number (CN) = 4: *r* = 0.99 Å for Na^+^ vs *r* = 0.59 Å for Li^+^).^46^ However, despite the significantly larger ionic radius, we found that Na^+^ coordinates to the Me_6_Tren and DETAN ligands in a similar manner to Li^+^. Like LiI, sodium iodide (NaI) reacted with Me_6_Tren and DETAN to produce [Na(I)(κ^4^-*N*,*N*′,*N*″,*N*′′′-Me_6_Tren)] (**6**) and [Na(I)(DETAN)] (**7**), respectively (Scheme 3). Note that the sodium complexes **6** and **7** are more challenging to crystallize than the corresponding Li complexes: only **6** was obtained as single crystals suitable for the SCXRD study. Despite a decent crystalline yield (76%), we could only obtain microcrystals of **7**, which are too small even for the synchrotron X-ray source. On the other hand, the ^1^H and ^13^C{^1^H} NMR spectrum of **7** is similar to its lithium analogue **2** (Figures S5/S7 in the Supporting Information for **2**, Figures S17/S20 in the Supporting Information for **7**; Figure S19 in the Supporting Information for comparisons). To further confirm the monomeric structure of **7**, we conducted comparative diffusion-ordered NMR spectroscopy (DOSY)^54^ studies of **7** and its SCXRD-characterized Li analogue **2**. The protocols reported by Neufeld and Stalke^55^ were employed to determine the diffusion coefficient (*D*), which reflects the hydrodynamic radius of a molecule in the solution (see ref (55) and the Supporting Information for details). During the studies, adamantane was employed as the internal standard.^55^ For the monomeric [Li(I)(κ^4^-*N*,*N*′,*N*″,*N*′′′-DETAN)] (**2**), the *D* value is 9.89 × 10^–10^ m^2^ s^–1^; while for **7**, the *D* value is 9.93 × 10^–10^ m^2^ s^–1^. The similar *D* values confirm that **2** and **7** have similar hydrodynamic radii, i.e., they are both monomers in solution.

**Figure 4:**
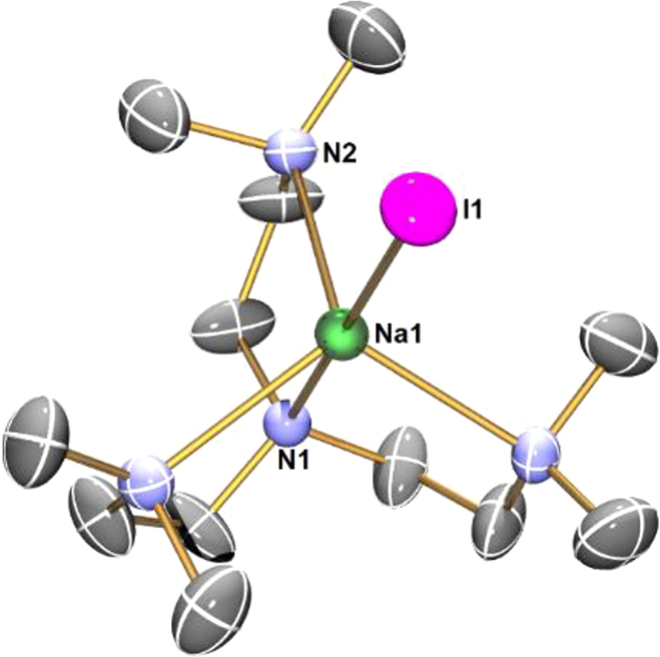
X-ray crystal structure of [Na(I)(κ^4^-*N*,*N*′,*N*″,*N*′′′-Me_6_Tren)] (**6**) at 150 K with 50% probability ellipsoids. Hydrogen atoms are omitted for the sake of clarity. Only crystallographically independent noncarbon atoms are labeled. The selected bond distances are as follows: Na1–N1, 2.490(4) Å; Na1–N2, 2.462(8) Å; Na1–I1, 3.004(6) Å. The selected bond angles are as follows: N1–Na1–I1, 180.0°; N1–Na1–N2, 73.6(2)°; N2–Na1–I1, 106.4(2)°. The atomic color codes: Na (forest green); C (gray); N (blue); I (magenta).

The single-crystal structure of **6** is exhibited in Figure 4. Structural comparison between **6** and its Li analogue **1** would be informative. Both **1** and **6** have a trigonal bipyramidal coordination geometry, but the Na–N and Na–I bonds in **6** are ca. 0.4 Å longer than the corresponding Li–N and Li–I bonds in **1**, which reflects the larger Na^+^ ionic radius.^46^ As a result, in **6**, the Na^+^ cation sits above the plane defined by the three equatorial N atoms by 0.70 Å. In comparison, in **1**, the Li^+^ cation is much more in the plane with an out-of-plane distance of 0.32 Å.

**Scheme 4:**
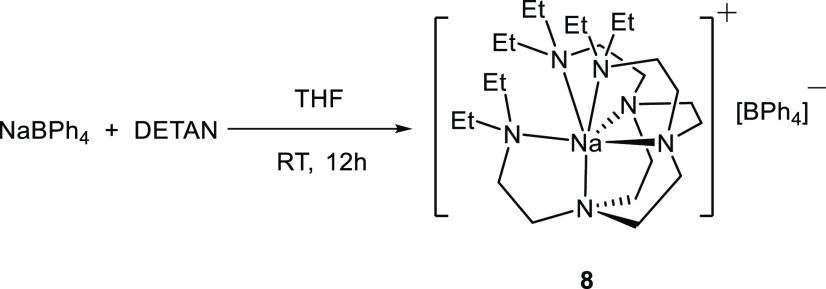
Reaction To Produce [Na(κ^6^*-N*6-DETAN)][BPh_4_] (**8**)

**Figure 5:**
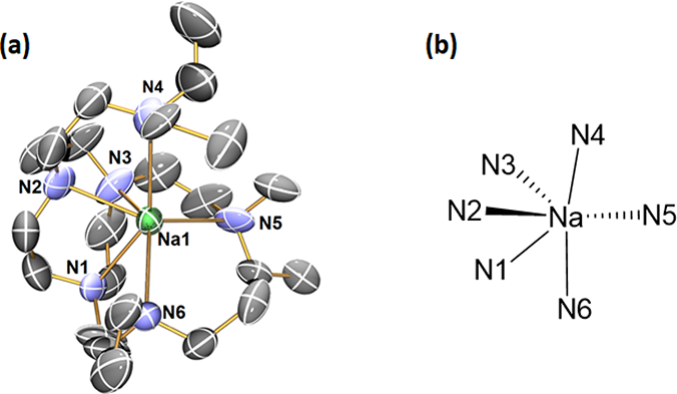
(a) X-ray crystal structure of [Na(κ^6^-*N*6-DETAN)][BPh_4_] (**8**) at 150 K with 50% probability ellipsoids. Hydrogen atoms, the minor disorder component and the [BPh_4_]^−^ anion are omitted for the sake of clarity. (b) The schematic representation of the distorted trigonal prismatic coordination geometry of the Na^+^ center. The selected bond distances are as follows: Na1–N1, 2.480(7) Å; Na1–N2 2.471(8) Å; Na1–N3 2.459(9) Å; Na1–N4 2.892(8) Å; Na1–N5 2.747(8) Å; Na1–N6 2.798(8) Å. The selected bond angles are as follows: N1–Na1–N2, 72.8(2)°; N2–Na1–N3, 73.5(3)°; N3–Na1–N4, 139.4(3)°; N4–Na1–N5, 106.6(3)°; N5–Na1–N6, 111.4(3)°; N6–Na1–N1, 100.4(3)°. [Atomic color codes: Li (forest green); C (gray); N (blue).]

The reaction between NaBPh_4_ and DETAN produced a white crystalline product (**8**) (Scheme 4). Complex **8** is a DETAN-coordinated separated ion-pair complex. The cation [Na(κ^6^-DETAN)]^+^ features a six-coordinate Na^+^ center (Figure 5a): all the six N atoms of the DETAN ligand coordinate, forming a distorted trigonal prismatic geometry (Figure 5b). This is in sharp contrast with the four-coordinate trigonal pyramidal [Li(κ^4^-*N*,*N*′,*N*″,*N*′′′-DETAN)]^+^ cation in complex **5** (Figure 3b). The six Na–N bonds in **8** divided into two groups: (1) the N atoms in the macrocycle (N_cyc_) coordinate to the Na^+^ via short Na–N dative bonds, ca. 2.47 Å (Na–N1/N2/N3); (2) the N atoms in the side arms (N_arm_) coordinate to the Na^+^ via long Na–N dative bonds, ca. 2.80 Å (Na–N4/N5/N6). However, even the longer Na–N_arm_ bonds are much shorter than the weakly coordinated Li**···**N distance (3.022 Å) in **5**. We attribute the structural differences between the [Li(κ^4^-*N*,*N*′,*N*″,*N*′′′-DETAN)]^+^ (**5**) and the [Na(κ^6^-DETAN)]^+^ (**8**) to their different ionic radii of Li^+^ (*r* = 0.59 Å, CN = 4) and Na^+^ (*r* = 1.02 Å, CN = 6).^46^

**Scheme 5:**
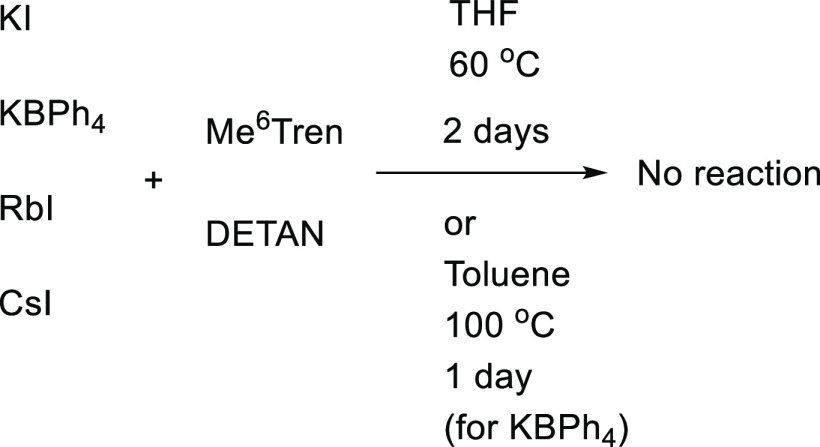
K^+^/Rb^+^/Cs^+^ Iodides/Tetraphenylborates Do Not React with the DETAN/Me_6_Tren Ligands

Since the DETAN and Me_6_Tren ligands exhibited versatile coordination chemistry to Li and Na cations as demonstrated in the complexes **1**–**8**, we tested their coordinating reactions with larger Group 1 metal halides and tetraphenylborates, i.e., K^+^, Rb^+^, and Cs^+^. However, the reactions between a variety of K^+^/Rb^+^/Cs^+^ reagents (KI, KBPh_4_, RbI, CsI) and the DETAN/Me_6_Tren ligands did not proceed at room temperature nor 60 °C within 2 days (Scheme 5). Harsher conditions were examined for KBPh_4_, which is supposed to be the most reactive one among the K/Rb/Cs substrates for its small K^+^ cation and more-likely soluble BPh_4_^–^ anion. After heating at 100 °C in toluene for 24 h, there was no reaction between KBPh_4_ and 1 equiv of Me_6_Tren or DETAN. We attribute the inertness of these larger Group 1 metal iodides/tetraphenylborates to two possible reasons: (1) their larger ionic radii may not be suitable for the ligands, although there were reports of K^23^/Rb^56^/Cs^56,57^ Me_6_Tren complexes; (2) the MX’s poor solubility (where M = K, Rb, Cs; X = I, BPh_4_) in THF/toluene may prevent the reactions, although the LiX/NaX are comparably insoluble in the above-mentioned solvents.

## Results and Discussion

The coordination modes of the Li^+^/Na^+^ DETAN/Me_6_Tren complexes are summarized in Table 1. The multidentate neutral Me_6_Tren and DETAN ligands coordinate to Li^+^ (*r* = 0.59 Å, CN = 4) and Na^+^ (*r* = 0.99 Å, CN = 4), exhibiting versatile coordination modes, from trigonal bipyramidal (**1**, **2**, **6**), tetrahedral (**3**), trigonal pyramidal (**4**, **5**) to trigonal prismatic (**8**) (Table 1). The coordination mode is dependent on the metal ionic radii and the steric profile of the metal center. Complexes with less steric congestion, i.e., longer Li–E bond and smaller E groups, such as the Li–I complexes **1** and **2**, have a tendency to form five-coordinated trigonal bipyramidal geometry, while the side arms of the Me_6_Tren or DETAN remain coordinated. The larger Li–E groups, such as −CH_3_ (**3**) and −CH_2_SiMe_3_ (**4**), have a tendency to cause the decoordination of the side arms. Without any E group, the cationic Li^+^ center, on the other hand, formed an N-capped four-coordinate trigonal pyramidal geometry in complex **5**, instead of a five-coordinated trigonal bipyramidal geometry, probably due to the geometric strains of the DETAN ligand. In comparison, the larger Na^+^ cation forms a six-coordinate distorted trigonal prismatic geometry in complex **8**, where all the six N atoms of the DETAN ligand coordinate.

**Table 1:**
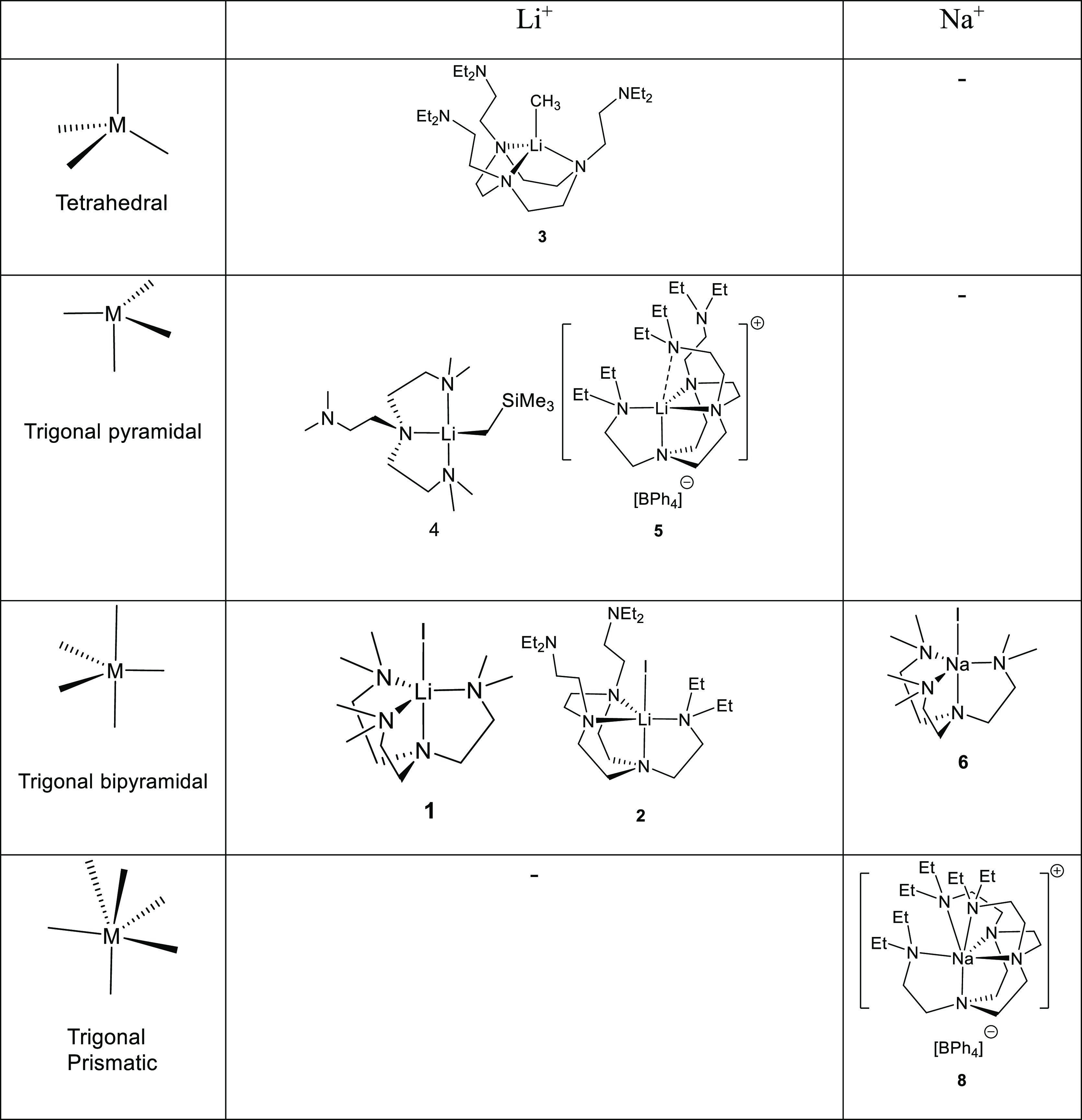
Diversified Solid-State Coordination Modes of the Me_6_Tren/DETAN Ligands with Li^+^/Na^+^

We would like to draw our reader’s attention to the fact that all the previous discussions about coordination modes are based on the SCXRD structures, i.e., in solid state. In solutions, the situations could be more complicated. The discrepancy between solid-state and solution-state structures is a long-lasting debateful topic in coordination chemistry, particularly in s-block metal chemistry.^58,59^ It is possible that the DETAN and Me_6_Tren ligands exhibit a rapid coordination/decoordination equilibrium, involving the entire ligand or a part of it. This hypothesis is proved by deliberately introducing an extra amount of free DETAN ligand to the isolated [Na(I)(DETAN)] (**7**). The ^1^H NMR spectrum of a mixture of [Na(I)(DETAN)] (**7**) (0.0288 g, 0.05 mmol), free DETAN ligand (0.0085 g, 0.02 mmol), and an internal NMR integration standard adamantane (0.0068 g, 0.05 mmol) (see Figure S20) exhibit new ^1^H NMR signals, which are different from both the free DETAN and **7**. However, the complicated solution-state behaviors will not compromise our previous solid-state structural discussions. A comprehensive and systematic solution-state NMR study (including temperature-/concentration-dependent behaviors) about this series of DETAN/Me_6_Tren complexes, which would complement this work, is out of the scope of this Article and will be published in short due.

## Conclusions and Outlook

This work demonstrated the capability and versatility of two multidentate neutral amine ligands: the *tetra*-dentate Me_6_Tren and the *hexa*-dentate DETAN, in Group 1 metal chemistry. The side arms of the Me_6_Tren/DETAN ligands exhibited flexible coordinating capabilities, which could act as on-demand internal Lewis bases to promote desired reactions and to compensate the reduction of coordination numbers upon the formation of a desired highly reactive species, such as from a heteroleptic metal alkyl amide complex to a terminal metal imido complex via alkane elimination.^37,39^

As a closing remark, this work will serve as a guidebook for future adventures employing the DETAN/Me_6_Tren ligands to isolate highly reactive Group 1 and Group 2 metal complexes and their reactivity studies, such as small molecular activations. Given the similar ionic M–E bonding character, the knowledge of DETAN’s coordination modes could also be extrapolated into rare-earth metal chemistry, to pursue high-value targets such as divalent rare-earth terminal imides^37^ and phosphinidenes.^60^ This work could also help the coordination chemistry and catalysis communities to choose suitable multidentate neutral amine ligands to design new metal complexes and stoichiometric/catalytic reactions.

## Supporting Information Available

Synthesis and characterizations of the complexes **1**, **2**, and **5**–**8**, as well as the calculations (PDF)

## Accession Codes

CCDC 2120222–2120225 and 2125503 contain the supplementary crystallographic data for this paper. These data can be obtained free of charge via www.ccdc.cam.ac.uk/data_request/cif, or by emailing data_request@ccdc.cam.ac.uk, or by contacting The Cambridge Crystallographic Data Centre, 12 Union Road, Cambridge CB2 1EZ, UK; fax: +44 1223 336033.

## References

[ref1] King D. M.; Tuna F.; McInnes E. J. L.; McMaster J.; Lewis W.; Blake A. J.; Liddle S. T. Synthesis and structure of a terminal uranium nitride complex. Science 2012, 337 (6905), 717–720. 10.1126/science.1223488. 22745250

[ref2] Légaré M. – A.; Bélanger-Chabot G.; Dewhurst R. D.; Welz E.; Krummenacher I.; Engels B.; Braunschweig H. Nitrogen fixation and reduction at boron. Science 2018, 359 (6378), 896–900. 10.1126/science.aaq1684. 29472479

[ref3] Auerhammer D.; Arrowsmith M.; Dewhurst R. D.; Kupfer T.; Böhnke J.; Braunschweig H. Closely related yet different: a borylene and its dimer are non-interconvertible but connected through reactivity. Chem. Sci. 2018, 9, 2252–2260. 10.1039/C7SC04789D. 29719698 PMC5897878

[ref4] Arrowsmith M.; Braunschweig H.; Celik M. A.; Dellermann T.; Dewhurst R. D.; Ewing W. C.; Hammond K.; Kramer T.; Krummenacher I.; Mies J.; Radacki K.; Schuster J. K. Neutral zero-valent s-block complexes with strong multiple bonding. Nat. Chem. 2016, 8, 890–894. 10.1038/nchem.2542. 27334631

[ref5] Wang G. – C.; Walley J. E.; Dickie D. A.; Pan S.; Frenking G.; Gilliard R. J. Jr A Stable, Crystalline Beryllium Radical Cation. J. Am. Chem. Soc. 2020, 142 (10), 4560–4564. 10.1021/jacs.9b13777. 32088963

[ref6] The Chemistry of Organolithium Compounds; Rappoport Z., Mare I., Eds; John Wiley & Sons, Ltd., Chichester, West Sussex, England, 2004.

[ref7] Klett J. Structural Motifs of Alkali Metal Superbases in Non-coordinating Solvents. Chem.—Eur. J. 2021, 27, 888–904. 10.1002/chem.202002812. 33165981 PMC7839563

[ref8] Hong L.; Sun W. – S.; Yang D. – X.; Li G. – F.; Wang R. Additive Effects on Asymmetric Catalysis. Chem. Rev. 2016, 116, 4006–4123. 10.1021/acs.chemrev.5b00676. 26881289

[ref9] Kurono N.; Yamaguchi M.; Suzuki K.; Ohkuma T. Lithium Chloride: An Active and Simple Catalyst for Cyanosiylation of Aldehydes and Ketones. J. Org. Chem. 2005, 70, 6530–6532. 10.1021/jo050791t. 16050725

[ref10] Hopkinson M. N.; Richter C.; Schedler M.; Glorius F. An overview of N-heterocyclic carbenes. Nature 2014, 510, 485–496. 10.1038/nature13384. 24965649

[ref11] Soleilhavoup M.; Bertrand G. Cyclic (Alkyl)(Amino)Carbenes (CAACs): Stable Carbenes on the Rise. Acc. Chem. Res. 2015, 48, 256–266. 10.1021/ar5003494. 25515548

[ref12] Vögtle F.; Weber E. Multidentate Acyclic Neutral Ligands and Their Complexation. Angew. Chem., Int. Ed. Engl. 1979, 18, 753 10.1002/anie.197907531.

[ref13] Reich H. J. Role of Organolithium Aggregates and Mixed Aggregates in Organolithium Mechanisms. Chem. Rev. 2013, 113, 7130–7178. 10.1021/cr400187u. 23941648

[ref14] Harrison-Marchand A.; Mongin F. Mixed AggregAte (MAA): A Single Concept for All Dipolar Organometallic Aggregates. 1. Structural Data. Chem. Rev. 2013, 113, 7470–7562. 10.1021/cr300295w. 23952819

[ref15] Mongin F.; Harrison-Marchand A. Mixed AggregAte (MAA): A Single Concept for All Dipolar Organometallic Aggregates. 2. Syntheses and Reactivities of Homo/HeteroMAAs. Chem. Rev. 2013, 113, 7563 10.1021/cr3002966. 23952912

[ref16] Gentner T. X.; Mulvey R. E. Alkali-Metal Mediation: Diversity of Applications in Main-Group Organometallic Chemistry. Angew. Chem., Int. Ed. 2021, 60, 9247–9262. 10.1002/anie.202010963. PMC8247348 33017511

[ref17] Collum D. B. Is *N,N,N’,N’*-tetramethylethylenediamine a good ligand for lithium?. Acc. Chem. Res. 1992, 25, 448–454. 10.1021/ar00022a003.

[ref18] Strohmann C.; Seibel T.; Strohfeldt K. [*t*BuLi·(−)-Sparteine]: Molecular Structure of the First Monomeric Butyllithium Compound. Angew. Chem., Int. Ed. 2003, 42, 4531–4533. 10.1002/anie.200351308. 14520758

[ref19] Knauer L.; Wattenberg J.; Kroesen U.; Strohmann C. The smaller, the better? How the aggregate size affects the reactivity of (trimethylsilyl)methyllithium. Dalton Trans 2019, 48, 11285–11291. 10.1039/C9DT02182E. 31268108

[ref20] Reich H. J.; Green D. P.; Medina M. A.; Goldenberg W. S.; Gudmundsson B. Ö.; Dykstra R. R.; Phillips N. H. Aggregation and Reactivity of Phenyllithium Solutions. J. Am. Chem. Soc. 1998, 120, 7201–7210. 10.1021/ja980684z.

[ref21] Raston C. L.; Whitaker C. R.; White A. H. Lewis-Base Adducts of Main Group Metal(I) Compounds. XI. Di-μ-iodo-bis(N,N,N′N′′,N′′-pentamethyldiethylenetriamine-N,N′,N′′-sodium). Aust. J. Chem. 1989, 42, 1393–1396. 10.1071/CH9891393.

[ref22] Cousins D. M.; Davidson M. G.; Frankis C. J.; García-Vivó D.; Mahon M. F. Tris(2-dimethylaminoethyl)amine: A simple new tripodal polyamine ligand for Group 1 metals. Dalton Trans 2010, 39, 8278–8280. 10.1039/c0dt00567c. 20689868

[ref23] Davidson M. G.; García-Vivó D.; Kennedy A. R.; Mulvey R. E.; Robertson S. D. Exploiting σ/π Coordination Isomerism to Prepare Homologous Organoalkali Metal (Li, Na, K) Monomers with Identical Ligand Sets. Chem.—Eur. J. 2011, 17, 3364–3369. 10.1002/chem.201003493. 21341333

[ref24] Armstrong D. R.; Davidson M. G.; García-Vivó D.; Kennedy A. R.; Mulvey R. E.; Robertson S. D. Monomerizing Alkali-Metal 3,5-Dimethylbenzyl Salts with Tris(N, N-dimethyl-2-aminoethyl)amine (Me_6_TREN): Structural and Bonding Implications. Inorg. Chem. 2013, 52, 12023–12032. 10.1021/ic401777x. 24088059

[ref25] Kennedy A. R.; Mulvey R. E.; Urquhart R. I.; Robertson S. D. Lithium, sodium and potassium picolyl complexes: syntheses, structures and bonding. Dalton Trans 2014, 43, 14265–14274. 10.1039/C4DT00808A. 24770550

[ref26] Robertson S. D.; Kennedy A. R.; Liggat J. J.; Mulvey R. E. Facile synthesis of a genuinely alkane-soluble but isolable lithium hydride transfer reagent. Chem. Commun. 2015, 51, 5452–5455. 10.1039/C4CC06421F. 25236757

[ref27] Leich V.; Spaniol T. P.; Okuda J. Formation of α-[KSiH_3_] by hydrogenolysis of potassium triphenylsilyl. Chem. Commun. 2015, 51, 14772–14774. 10.1039/C5CC06187C. 26299566

[ref28] Mukherjee D.; Osseili H.; Spaniol T. P.; Okuda J. Alkali Metal Hydridotriphenylborates [(L)M][HBPh_3_] (M = Li, Na, K): Chemoselective Catalysts for Carbonyl and O_2_ Hydroboration. J. Am. Chem. Soc. 2016, 138, 10790–10793. 10.1021/jacs.6b06319. 27513460

[ref29] Kennedy A. R.; McLellan R.; McNeil G. J.; Mulvey R. E.; Robertson S. D. Tetraamine Me_6_Tren induced monomerization of alkali metal borohydrides and aluminohydrides. Ployhedron 2016, 103, 94–99. 10.1016/j.poly.2015.08.046.

[ref30] Osseili H.; Mukherjee D.; Beckerle K.; Spaniol T. P.; Okuda J. Me_6_TREN-Supported Alkali Metal Hydridotriphenylborates [(L)M][HBPh_3_] (M = Li, Na, K): Synthesis, Structure, and Reactivity. Organometallics 2017, 36, 3029–3034. 10.1021/acs.organomet.7b00308.

[ref31] Davison N.; Waddell P. G.; Dixon C.; Wills C.; Penfold T. J.; Lu E. A monomeric (trimethylsilyl)methyl lithium complex: synthesis, structure, decomposition and preliminary reactivity studies. Dalton Trans. 2022, 10.1039/D1DT03532K. 34854445

[ref32] Standfuss S.; Spaniol T. P.; Okuda J. Lithiation of a Cyclen-Derived (NNNN) Macrocycle and Its Reaction with *n*-Butyllithium. Eur. J. Inorg. Chem. 2010, 2010, 2987–2991. 10.1002/ejic.201000199.

[ref33] Redko M. Y.; Jackson J. E.; Huang R. H.; Dye J. L. Design and Synthesis of a Thermally Stable Organic Electride. J. Am. Chem. Soc. 2005, 127, 12416–12422. 10.1021/ja053216f. 16131224

[ref34] Davison N.; Falbo E.; Waddell P. G.; Penfold T. J.; Lu E. A monomeric methyllithium complex: synthesis and structure. Chem. Commun. 2021, 57, 6205–6208. 10.1039/D1CC01420J. 34059860

[ref35] Fohlmeister L.; Stasch A. Alkali Metal Hydride Complexes: Well-Defined Molecular Species of Saline Hydrides. Aust. J. Chem. 2015, 68, 1190–1201. 10.1071/CH15206.

[ref36] Wolf B. M.; Anwander R. Chasing Multiple Bonding Interactions between Alkaline-Earth Metals and Main-Group Fragments. Chem.—Eur. J. 2019, 25, 8190–8202. 10.1002/chem.201901169. 30927501

[ref37] Wolf B. M.; Stuhl C.; Anwander R. Synthesis of homometallic divalent lanthanide organoimides from benzyl complexes. Chem. Commun. 2018, 54, 8826–8829. 10.1039/C8CC05234D. 30043800

[ref38] Wolf B. M.; Stuhl C.; Maichle-Mössmer C.; Anwander R. Lewis-Acid Stabilized Organoimide Complexes of Divalent Samarium, Europium, and Ytterbium. Chem.—Eur. J. 2018, 24, 15921–15929. 10.1002/chem.201803619. 30125407

[ref39] Lu E.; Chu J. – X.; Chen Y. – F. Scandium Terminal Imido Chemistry. Acc. Chem. Res. 2018, 51, 557–566. 10.1021/acs.accounts.7b00605. 29381048

[ref40] Raston C. L.; Skelton B. W.; Whitaker C. R.; White A. H. Lewis-Base Adducts of Main Group 1 Metal Compounds. IV. Synthesis and Structure of the XLiL_3_ System (X = Cl, Br, I, L = 4-t-Butylpyridine, and X = I, L = Quinoline). Aust. J. Chem. 1988, 41, 341 10.1071/CH9880341.

[ref41] Raston C. L.; Skelton B. W.; Whitaker C. R.; White A. H. Lewis-base adducts of main Group 1 metal compounds. Part 2. Syntheses and structures of [Li_4_Cl_4_(pmdien)_3_] and LiI(pmdien)]. J. Chem. Soc., Dalton Trans. 1988, 987–990. 10.1039/dt9880000987.

[ref42] Berthet J. – C.; Siffredi G.; Thuéry P.; Ephritikhine M. Synthesis and crystal structure of pentavalent uranyl complexes. The remarkable stability of UO_2_X (X = I, SO_3_CF_3_) in non-aqueous solutions. Dalton Trans. 2009, 3478 10.1039/b820659g. 19381410

[ref43] Liu F. – C.; Shadike Z.; Wang X. – F.; Shi S.-Q.; Zhou Y. – N.; Chen G. – Y.; Yang X. – Q.; Weng L. – H.; Zhao J. – T.; Fu Z. – W. A Novel Small-Molecule Compound of Lithium Iodine and 3-Hydroxypropionitride as a Solid-State Electrolyte for Lithium–Air Batteries. Inorg. Chem. 2016, 55, 6504–6510. 10.1021/acs.inorgchem.6b00564. 27308962

[ref44] Thirumoorthi R.; Chivers T. Structural Comparison of Lithium Iodide Complexes of Symmetrical and Unsymmetrical [CH_2_(PPh_2_NSiMe_3_)(PPh_2_NR)] (R = SiMe_3_, H) Ligands. J. Struct. Chem. 2018, 59, 1221–1227. 10.1134/S002247661805030X.

[ref45] Ivanova I. S.; Ilyukhin A. B.; Tsebrikova G. S.; Polyakova I. N.; Pyatova E. N.; Solov’ev V. P.; Baulin V. E.; Tsivadze A. Y. 2,4,6-Tris[2-(diphenylphosphoryl)-4-ethylphenoxy]-1,3,5-triazine: A new ligand for lithium binding. Inorg. Chim. Acta 2019, 497, 119095 10.1016/j.ica.2019.119095.

[ref46] Shannon R. D. Revised effective ionic radii and systematic studies of interatomic distances in halides and chalcogenides. Acta Crystallogr. 1976, A32, 751–767. 10.1107/S0567739476001551.

[ref47] Perdew J. P.; Ernzerhof M.; Burke K. Rationale for mixing exact exchange with density functional approximations. J. Chem. Phys. 1996, 105, 9982–9985. 10.1063/1.472933.

[ref48] Becke A. D. Density-functional thermochemistry. III. The role of exact exchange. J. Chem. Phys. 1993, 98, 5648–5652. 10.1063/1.464913.

[ref49] Lee C. T.; Yang W. T.; Parr R. G. Development of the Colle-Salvetti correlation-energy formula into a functional of the electron density. Phys. Rev. B 1988, 37, 785–789. 10.1103/PhysRevB.37.785. 9944570

[ref50] Dill J. D.; Pople J. A. Self-consistent molecular orbital methods. XV. Extended Gaussian-type basis sets for lithium, beryllium, and boron. J. Chem. Phys. 1975, 62, 2921–2923. 10.1063/1.430801.

[ref51] Weigend F.; Ahlrichs R. Balanced basis sets of split valence, triple zeta valence and quadruple zeta valence quality for H to Rn: Design and assessment of accuracy. Phys. Chem. Chem. Phys. 2005, 7, 3297–3305. 10.1039/b508541a. 16240044

[ref52] Mantina M.; Chamberlin A. C.; Valero R.; Cramer C. J.; Truhlar D. G. Consistent van der Waals Radii for the Whole Main Group. J. Phys. Chem. A 2009, 113, 5806–5812. 10.1021/jp8111556. 19382751 PMC3658832

[ref53] Pollard V. A.; Orr S. A.; McLellan R.; Kennedy A. R.; Hevia E.; Mulvey R. E. Lithium diamidodihydridoaluminates: bimetallic cooperativity in catalytic hydroboration and metalation applications. Chem. Commun. 2018, 54, 1233–1236. 10.1039/C7CC08214B. 29336450

[ref54] Barjat H.; Morris G. A.; Smart S.; Swanson A. G.; Williams S. C. R. High-Resolution Diffusion-Ordered 2D Spectroscopy (HR-DOSY) – A New Tool for the Analysis of Complex-Mixtures. J. Magn. Reson. Ser. B 1995, 108, 170–172. 10.1006/jmrb.1995.1118.

[ref55] Neufeld R.; Stalke D. Accurate molecular weight determination of small molecules *via* DOSY-NMR by using external calibration curves with normalized diffusion coefficients. Chem. Sci. 2015, 6, 3354–3364. 10.1039/C5SC00670H. 29142693 PMC5656982

[ref56] Du J.; Douair I.; Lu E.; Seed J. A.; Tuna F.; Wooles A. J.; Maron L.; Liddle S. T. Evidence for ligand- and solvent-induced disproporationation of uranium(IV). Nat. Commun. 2021, 12, 4832 10.1038/s41467-021-25151-z. 34376682 PMC8355312

[ref57] Ojeda-Amador A. I.; Martínez-Martínez A. J.; Kennedy A. R.; O’Hara C. T. Structural Studies of Cesium, Lithium/Cesium, and Sodium/Cesium Bid(trimethylsilyl)amide (HMDS) Complexes. Inorg. Chem. 2016, 55, 5719–5728. 10.1021/acs.inorgchem.6b00839. 27177080

[ref58] Woltornist R. A.; Collum D. B. Aggregation and Solvation of Sodium Hexamethyldisilazide: Across the Solvent Spectrum. J. Org. Chem. 2021, 86, 2406–2422. 10.1021/acs.joc.0c02546. 33471993 PMC8011853

[ref59] Neufeld R.; Stalke D. Solution Structure of Turbo-Hauser Base TMPMgCl·LiCl in d_8_-THF. Chem.—Eur. J. 2016, 22, 12624–12628. 10.1002/chem.201601494. 27224841

[ref60] Feng B.; Zhang H.-Y.; Qin H.; Peng Q.; Leng X.; Chen Y. Hydrogenation of Alkenes Catalyzed by Rare-Earth Metal Phosphinophosphinidene Complexes: 1,2-Addition/Elimination Versus σ-Bond Metathesis Mechanism. CCS Chem. 2021, 3, 3585–3594. 10.31635/ccschem.021.202101468.

