# Supplementary material for: Versatile Coordination Modes of Multidentate Neutral Amine Ligands with Group 1 Metal Cations
Source: Inorg Chem. 2022 Feb 11;61(8):3674–82. doi: 10.1021/acs.inorgchem.1c03786 (PMC9097481; doi:10.1021/acs.inorgchem.1c03786)
Supplement: Supplementary file 1 — ic1c03786_si_001.pdf [file ic1c03786_si_001.pdf]

# Supporting Information

## Versatile Coordination Modes of Multidentate Neutral Amine Ligands with Group-1 Metal Cations

Nathan Davison[a], Ke Zhou[c], Paul G. Waddell[a], Corrine Wills[a], Casey Dixon[a], Shu-Xian Hu[b*], Erli Lu[a*]

[a] *Chemistry-School of Natural and Environmental Sciences, Newcastle University. Newcastle upon Tyne, UK. NE1 7RU*

[b] *Beijing Computational Science Research Center. Beijing 100193, China.*

*c College of Chemistry and Environmental Science & Shannxi Key Laboratory of Catalysis & Institute of Theoretical and Computational Chemistry, Shannxi University of Technology. Hanzhong 723000, Shannxi Province, China.*

Corresponding authors: Erli Lu (erli.lu@newcastle.ac.uk); Shu-Xian Hu (hushuxian@csrc.ac.cn)

Contents

1. General procedures……..……………………………………….S2

2. Synthesis and Characterization of Complexes **1**, **2, 5**, **6-8**..…..…S3-S24

3. DOSY NMR Studies of complexes **2** and **7**…………………S24-S26

3. Single-Crystal X-Ray Crystallographic Data....…......…......……S27

4. Computational methods and data……………………………S28-S29

5. References....………………………………………………….S30

## General procedures

All manipulations were carried out using Schlenk techniques, or in a Vigor glovebox equipped with a -35 $^{o}C$ freezer and a cold well, under an atmosphere of dry argon. Solvents were dried by sodium metal and sodium/potassium alloy, then distilled under vacuum. All solvents were stored in the glovebox over potassium mirrors except for ethers, which were stored over activated 4 Å molecular sieves. The deuterated solvents were distilled from sodium/potassium alloy (for $d_6$-benzene and $d_8$-THF) or activated 4 Å molecular sieves (for $CDCl_3$), degassed by three freeze-pump-thaw cycles and stored under argon. *Tris*[2-(dimethylamino)ethyl]amine ($Me_6$Tren) was purchased from Merck and dried over 4 Å molecular sieves prior to use. *N,N',N"*-*Tris*-(2-*N*-diethylaminoethyl)-1,4,7-triaza-cyclononane (DETAN) was prepared as previously reported[1]. LiI, [$LiBPh_4(DME)_3$], NaI, $NaBPh_4$, KI, $KBPh_4$, RbI and CsI were purchased from Merck, Alfa Aesar and Strem, and dried under dynamic vacuum for 12 hours and stored in the glovebox prior to use.

$^{1}H$, $^{13}C\{^{1}H\}$ and $^{7}Li$ NMR spectra were recorded on a Bruker 300 Avance III spectrometer operating at 300.13, 75.48 and 116.64 MHz respectively. Chemical shifts are quoted in ppm and are relative to $SiMe_4$ ($^{1}H$ and $^{13}C$) or external 0.1M LiCl in $D_2O$ ($^{7}Li$).

Synthesis of [Li(I)($\kappa^4$-*N,N'N",N'"*-$Me_6$Tren)] (**1**)

$Me_6$Tren (0.691 g, 3 mmol) and LiI (0.402 g, 3 mmol) were combined in a 100 ml Schlenk flask. $Et_2O$ (40 ml) was added at room temperature and the resulting mixture was allowed to stir at room temperature for 20 hours, to afford a white suspension. The mixture was filtered, and the resulting white solid was washed with hexane (10 ml) and dried *in vacuo* to afford **1** as a white crystalline solid (1.05 g, 96% yield). Colourless needle-shaped single crystals suitable for SCXRD were obtained by slow cooling a dilute solution of **1** in boiling THF.

$^1$H NMR (300 MHz, $CDCl_3$, 25 °C): δ (ppm) 2.51 (m, 6H, N$CH_2CH_2$N), 2.37 (m, 6H, N$CH_2CH_2$N), 2.33 (s, 18H, N$CH_3$)$_2$)

$^{13}$C{$^1$H} NMR (75 MHz, $CDCl_3$, 25 °C): δ (ppm) 56.5 (N$CH_2CH_2$N), 49.9 (N$CH_2CH_2$N), 46.0 (N$CH_3$)$_2$)

$^7$Li NMR (117 MHz, $CDCl_3$, 25 °C): δ (ppm) 0.80

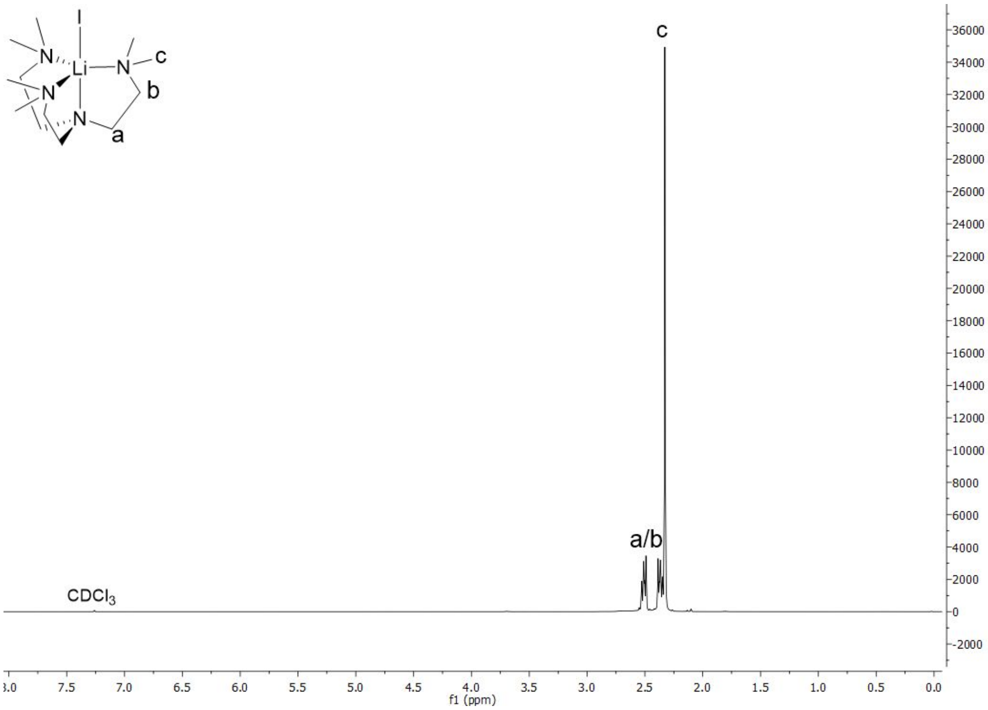


Figure S1. $^1$H NMR Spectrum of complex **1** [Li(I)($Me_6$Tren)].

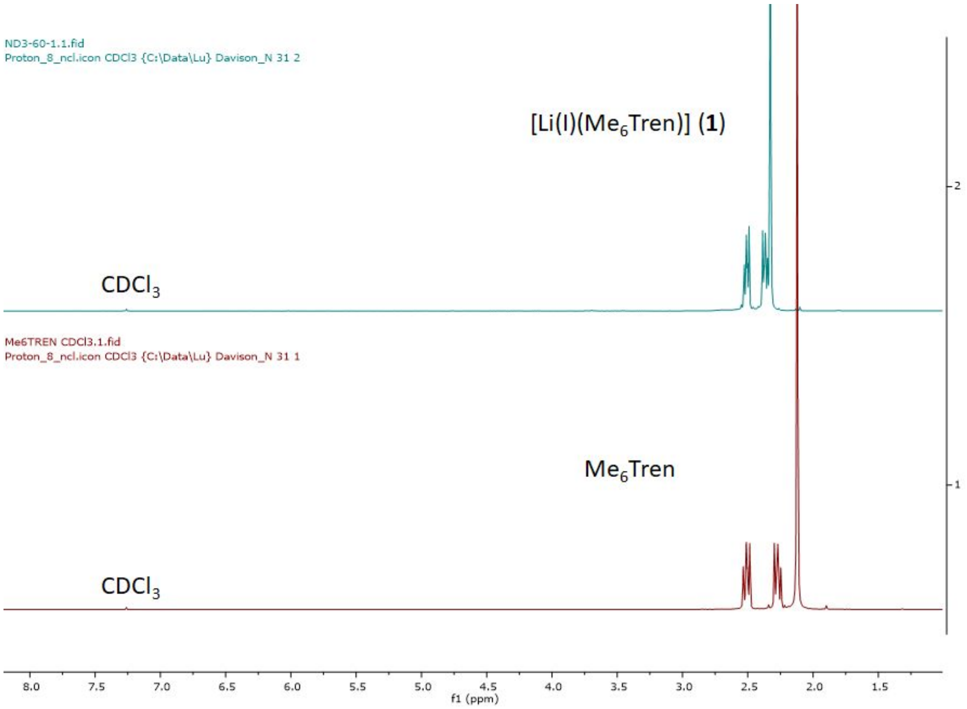


Figure S2. $^{1}$H NMR Spectra comparison between **1** and $Me_6$Tren in $CDCl_3$ at room temperature.

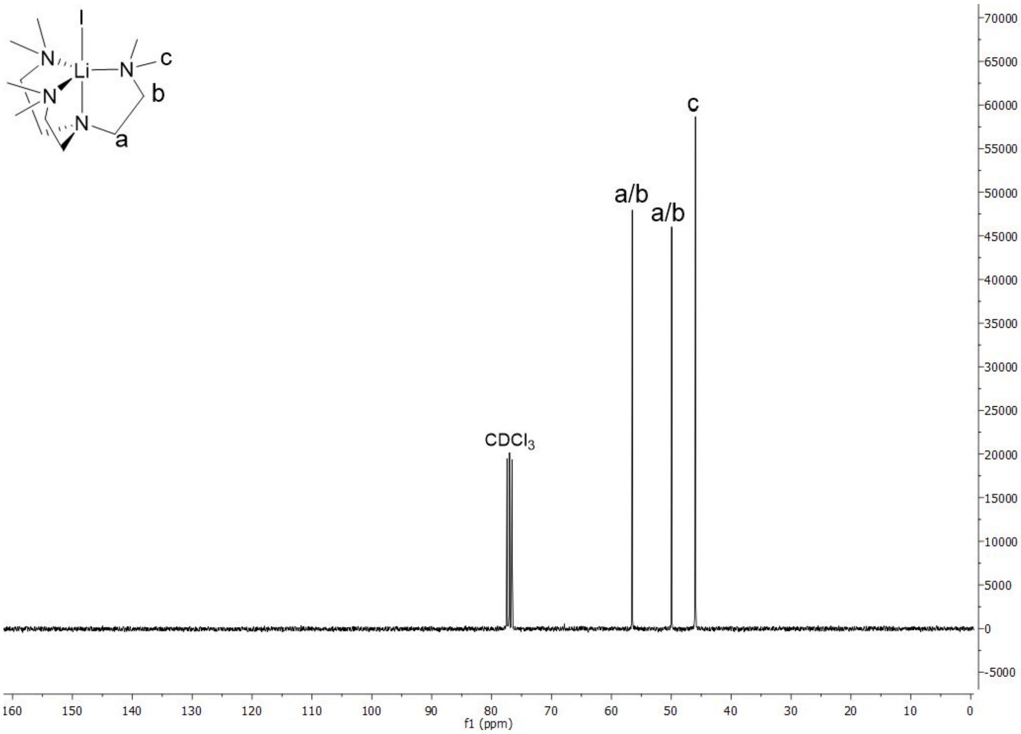


Figure S3. $^{13}C\{^{1}H\}$ NMR Spectrum of complex **1** [Li(I)($Me^6$Tren)].

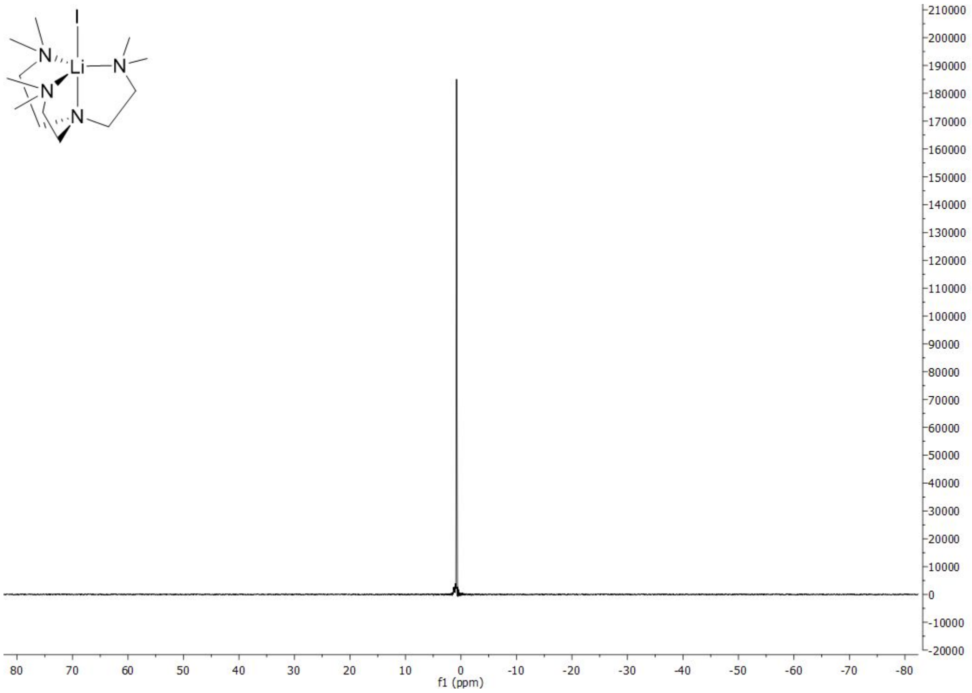

Figure S4. $^{7}$Li NMR Spectrum of complex **1** [Li(I)($Me^6$Tren)].

## Synthesis of [Li(I)(*κ*$^4$*-N,N'N",N'"*-DETAN)] (**2**)

DETAN (1.280 g, 3 mmol) and LiI (0.402 g, 3 mmol) were combined in a 100 ml Schlenk flask. $Et_2O$ (40 ml) was added at room temperature and the resulting solution was allowed to stir at room temperature for 20 hours, to afford a cloudy solution. The volatiles were removed *in vacuo* and the resulting white solid was washed with hexane (2 x 10 ml). All volatiles were removed under vacuum to afford **2** as a white crystalline solid (1.32 g, 79% yield). Single crystals suitable for SCXRD were obtained from a dilute $Et_2O$ solution (0.0073 g of **2** in 3 mL of $Et_2O$) after storing at -35 ˚C for 12 hours.

$^{1}$H NMR (500 MHz, $CDCl_3$, 25 °C): δ (ppm) 2.88 (m, 6H, N$CH_2$), 2.76 – 2.69 (m, 6H, N$CH_2$), 2.69 – 2.62 (m, 6H, N$CH_2$), 2.61 – 2.55 (m, 18H, N$CH_2$), 0.97 (t, $^{3}J_{HH}$ = 7.2 Hz, 18H, N$CH_2$$CH_3$).

$^{13}$C{$^{1}$H} NMR (126 MHz, $CDCl_3$, 25 °C): δ (ppm) 55.3 (N$CH_2$), 51.4 (N$CH_2$), 48.6 (N$CH_2$), 46.6 (N$CH_2$), 11.0 ($CH_2CH_3$).

$^{7}$Li NMR (194 MHz, $CDCl_3$, 25 °C): δ (ppm) 1.53 (minor), 1.42 (major), 1.31 (minor).

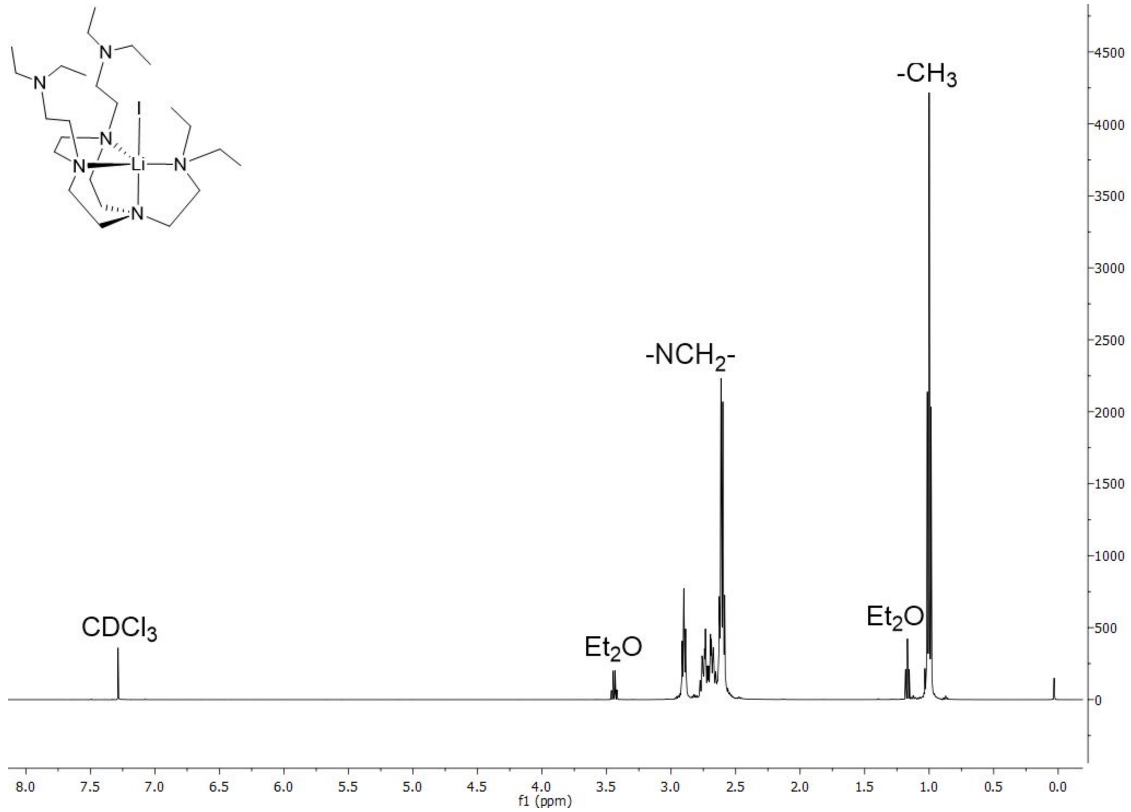


Figure S5. [1]H NMR Spectrum of complex **2** [Li(I)($\kappa^4$-*N,N'N'',N'''*-DETAN)].

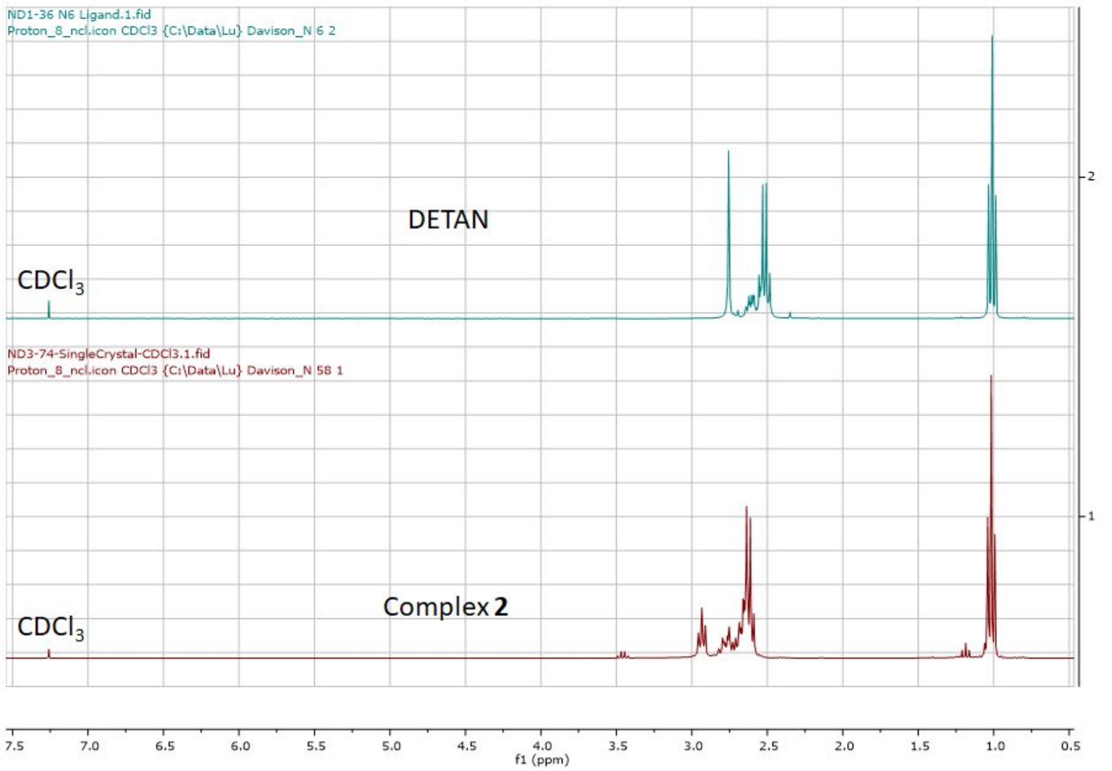

Figure S6. $^{1}H$ NMR Spectra comparison between **2** and DETAN in $CDCl_3$ at room temperature.

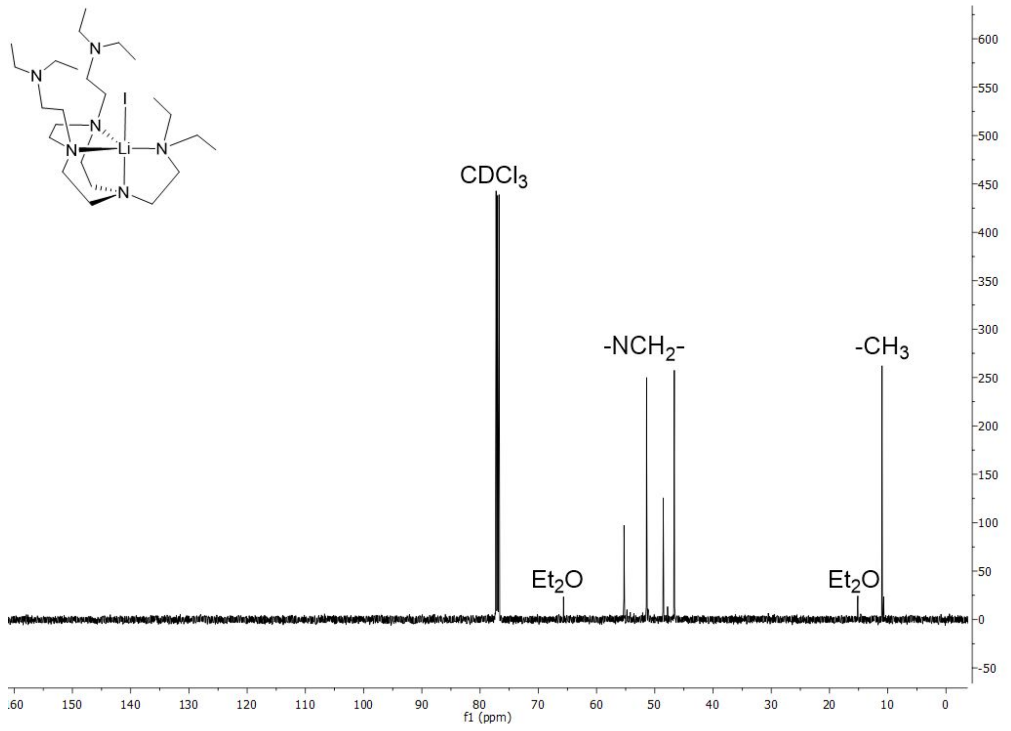


Figure S7. $^{13}C\{^{1}H\}$ NMR Spectrum of complex **2** [Li(I)($\kappa^4$-*N,N'N",N'"*-DETAN)].

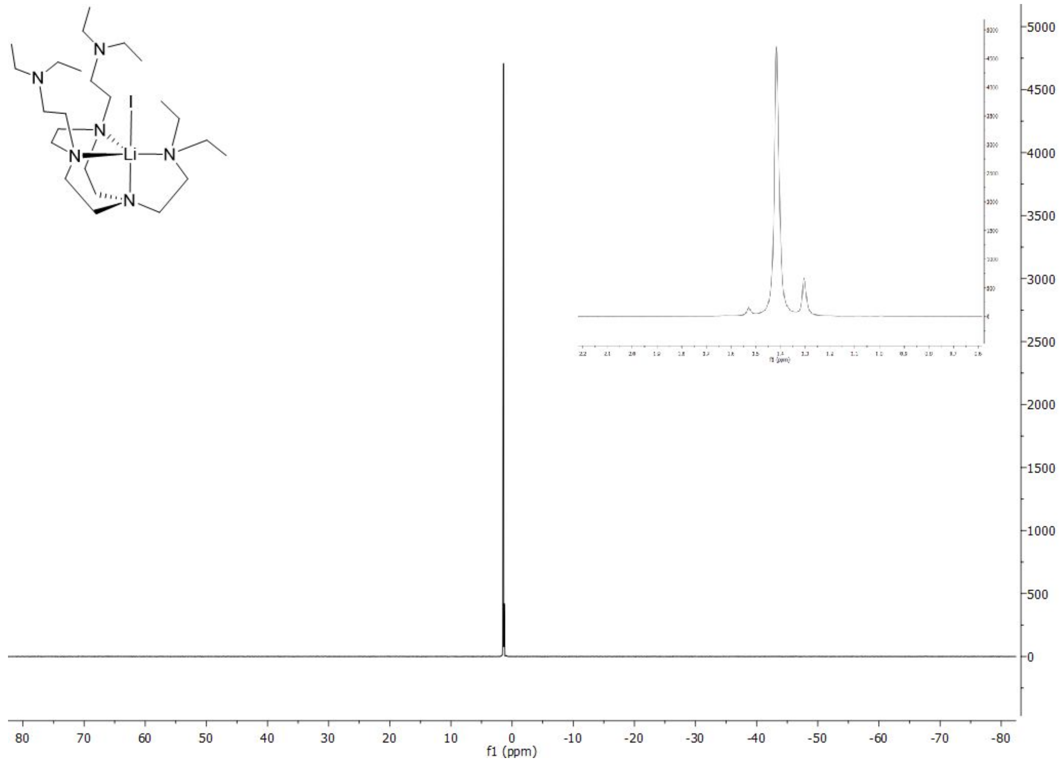


Figure S8. $^{7}$Li NMR Spectrum of complex **2** [Li(I)($\kappa^4$-*N,N'N",N'"*-DETAN)]. Inset: Zoom-in view of the $^{7}$Li signals to display the presence of the minor $^{7}$Li signals.

Synthesis of [Li($\kappa^3$-*N,N'N"*-DETAN)][$BPh_4$] (**5**)

At room temperature, a DETAN-$Et_2O$ solution (0.853 g DETAN, 2 mmol, in 10 mL of $Et_2O$) was added into a stirring solid of lithium tetraphenylborate *tris*(1,2-dimethoxyethane) adduct [$LiBPh_4(DME)_3$] (1.103 g, 2 mmol) in one-portion. THF (10 ml) was added to the mixture and the resulting colourless solution was stirred at room temperature for 18 hours. All volatiles were removed *in vacuo* and the resultant white solid was washed with hexane (10 mL) and dried *in vacuo*, to afford **5** as a white crystalline solid (1.26 g, 84% yield). Single crystals suitable for SCXRD were obtained from a $Et_2O$/THF (1 mL/0.2 mL) solution of **3** (0.376 g) at –35 °C.

$^{1}$H NMR (300 MHz, $CDCl_3$, 25 °C): δ (ppm) 7.42 (m, 8H, Ar-*H*s), 7.06 (t, $^{3}J_{HH}$ = 7.3 Hz, 8H, Ar-*H*s), 6.92 (m, 4H, Ar-*H*s), 2.64 – 2.46 (m, 18H, NC$H_2$), 2.46 – 2.37(m, 6H, NC$H_2$), 2.37 – 2.22 (m, 12H, NC$H_2$), 1.00 (t, $^{3}J_{HH}$ = 7.1 Hz, 18H, NCH$_2$C$H_3$).

$^{13}$C{$^{1}$H} NMR (75 MHz, $CDCl_3$, 25 °C): δ (ppm) 164.1 (q, $^{1}J_{BC}$ = 45.3 Hz, B-$C_{ipso}$), 136.2 (Ar-*C*s), 125.4 (Ar-*C*s), 121.6 (Ar-*C*s), 55.1, 51.7, 49.4 and 46.2 (N$CH_2$), 10.4 ($CH_2$$CH_3$).

$^{7}$Li NMR (117 MHz, $CDCl_3$, 25 °C): δ (ppm) 1.14

$^{11}$B NMR (96 MHz, $CDCl_3$, 25 °C): δ (ppm) -6.53

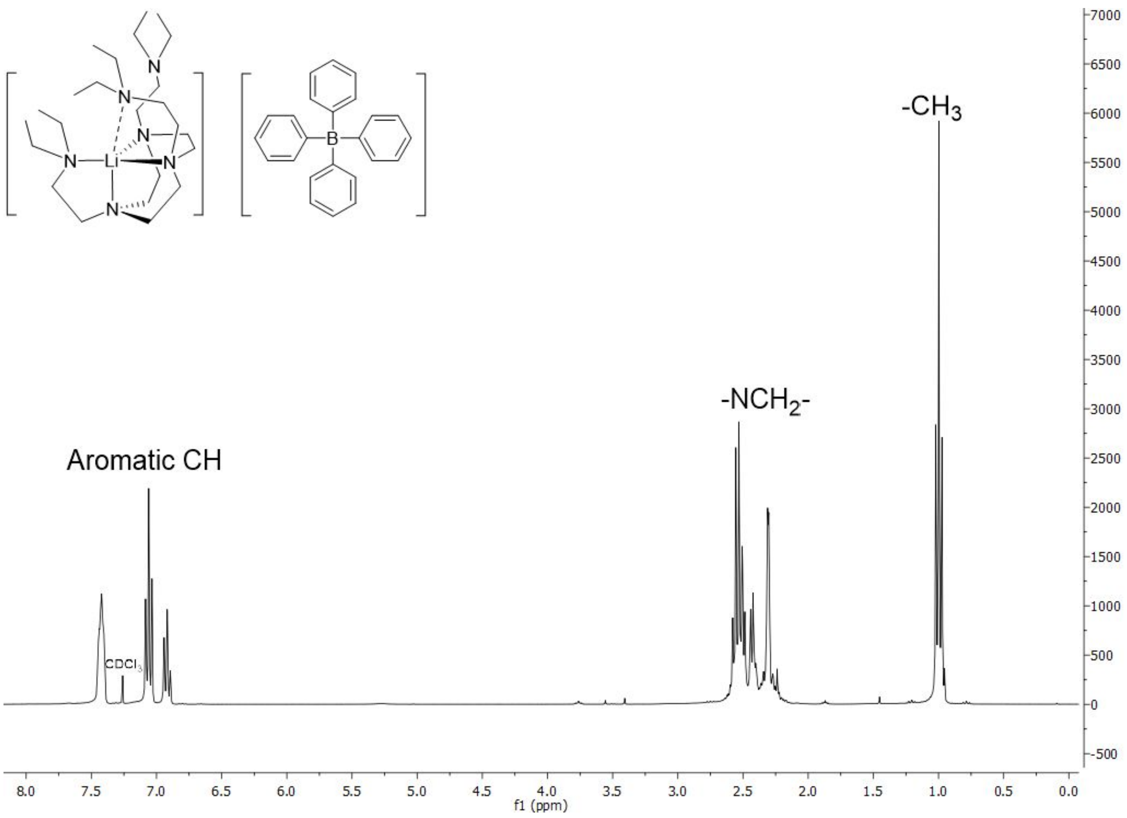


Figure S9. $^{1}$H NMR Spectrum of complex **5** [Li($\kappa^3$-*N,N'N''*-DETAN)][$BPh_4$].

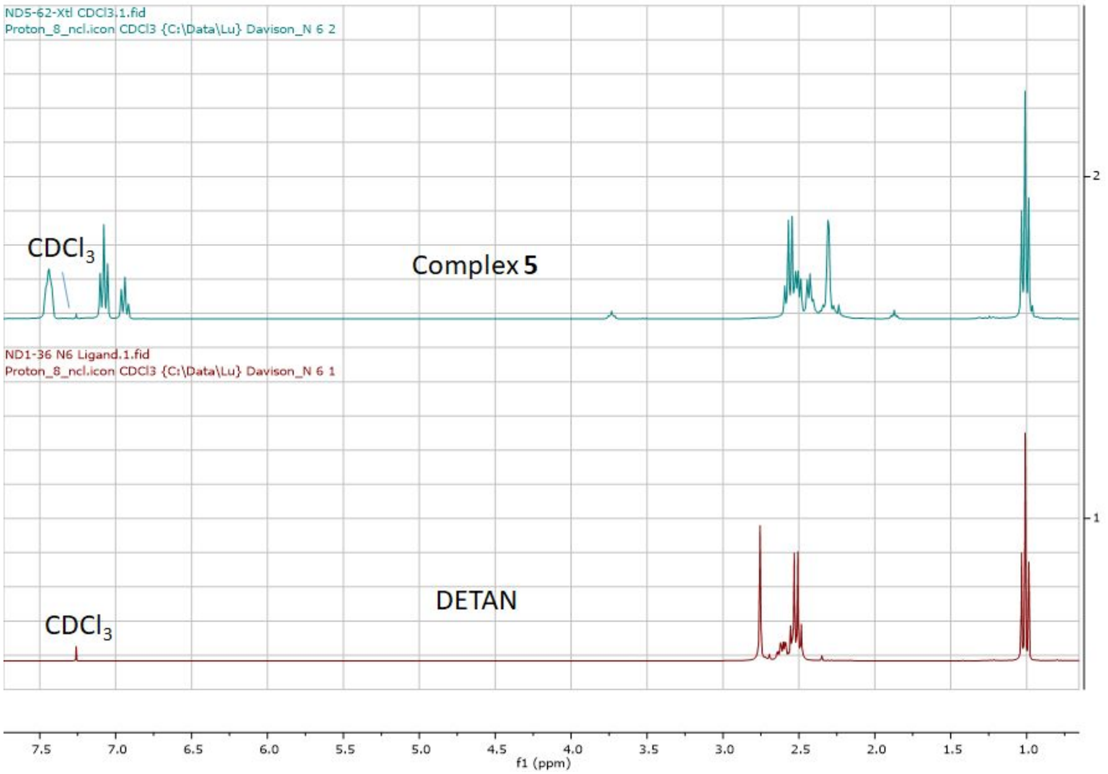


Figure S10. $^{1}$H NMR Spectra comparison between **5** and DETAN in $CDCl_3$ at room temperature.

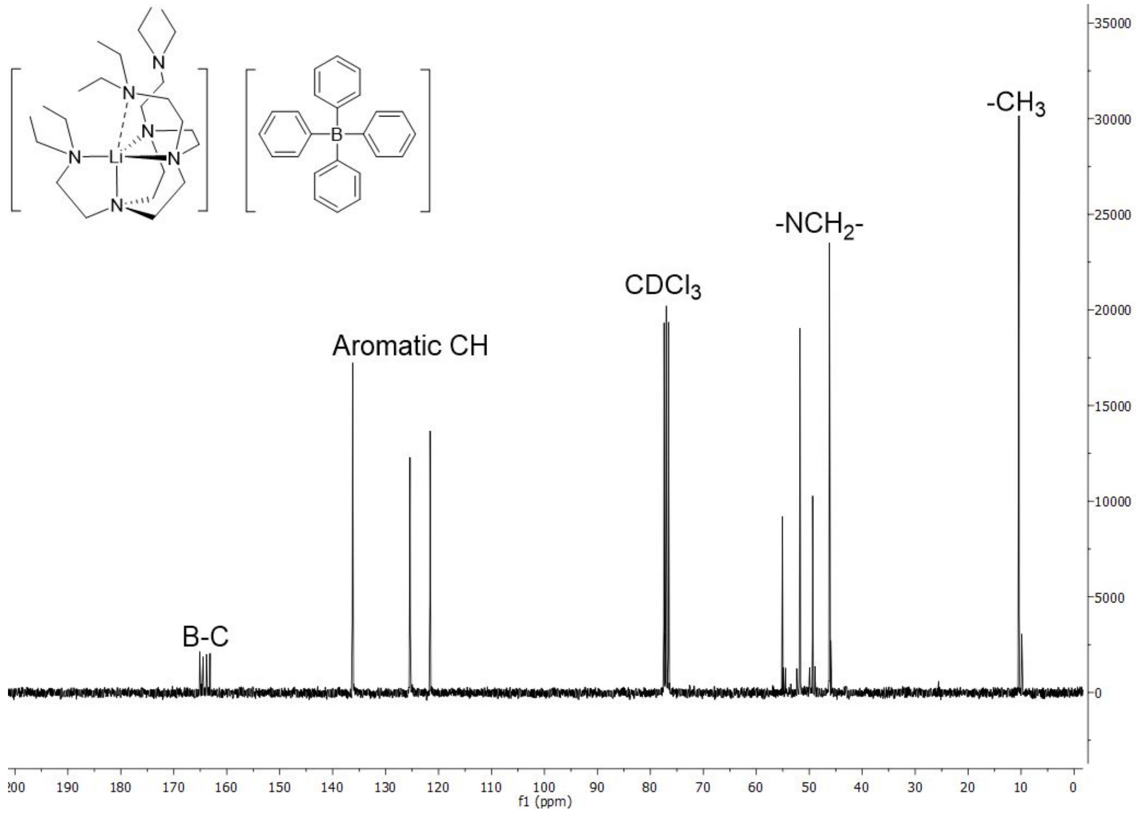


Figure S11. $^{13}C\{^{1}H\}$ NMR Spectrum of complex **5** [Li($\kappa^3$-*N,N',N''*-DETAN)][$BPh_4$].

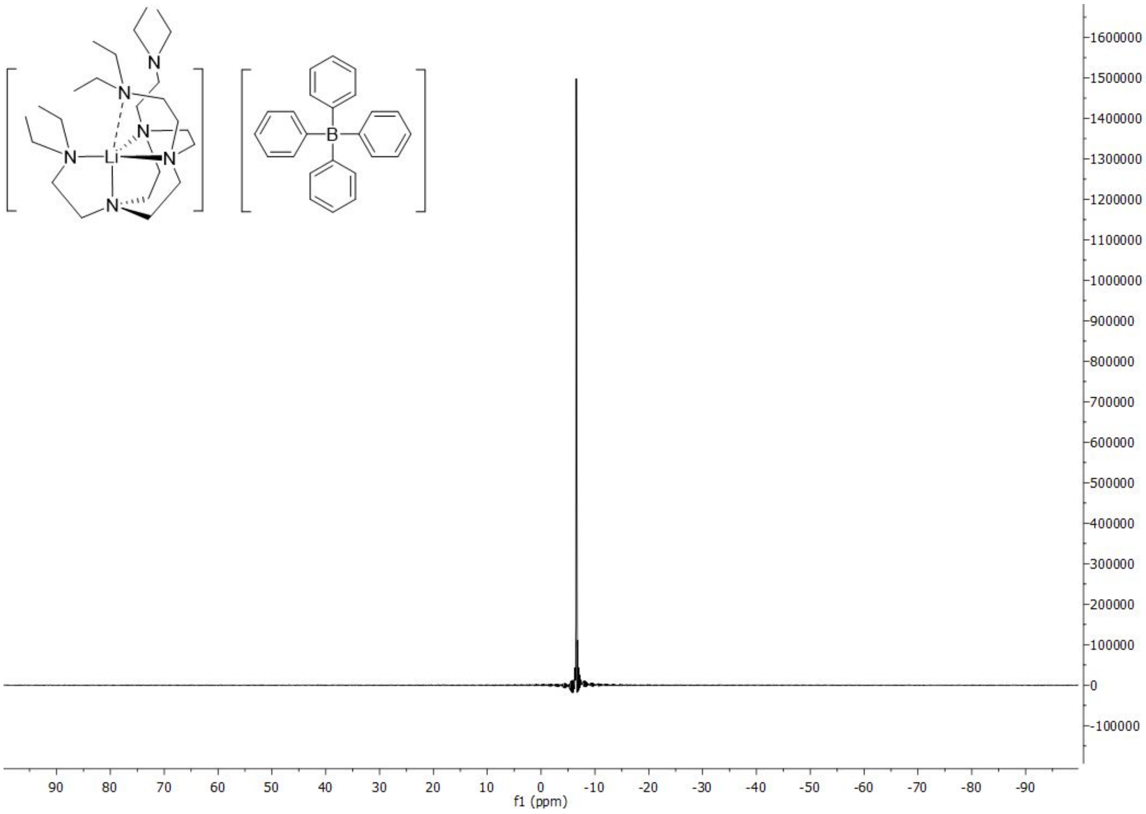

Figure S12. $^{11}$B NMR Spectrum of complex **5** [Li($\kappa^3$-*N,N',N''*-DETAN)][$BPh_4$].

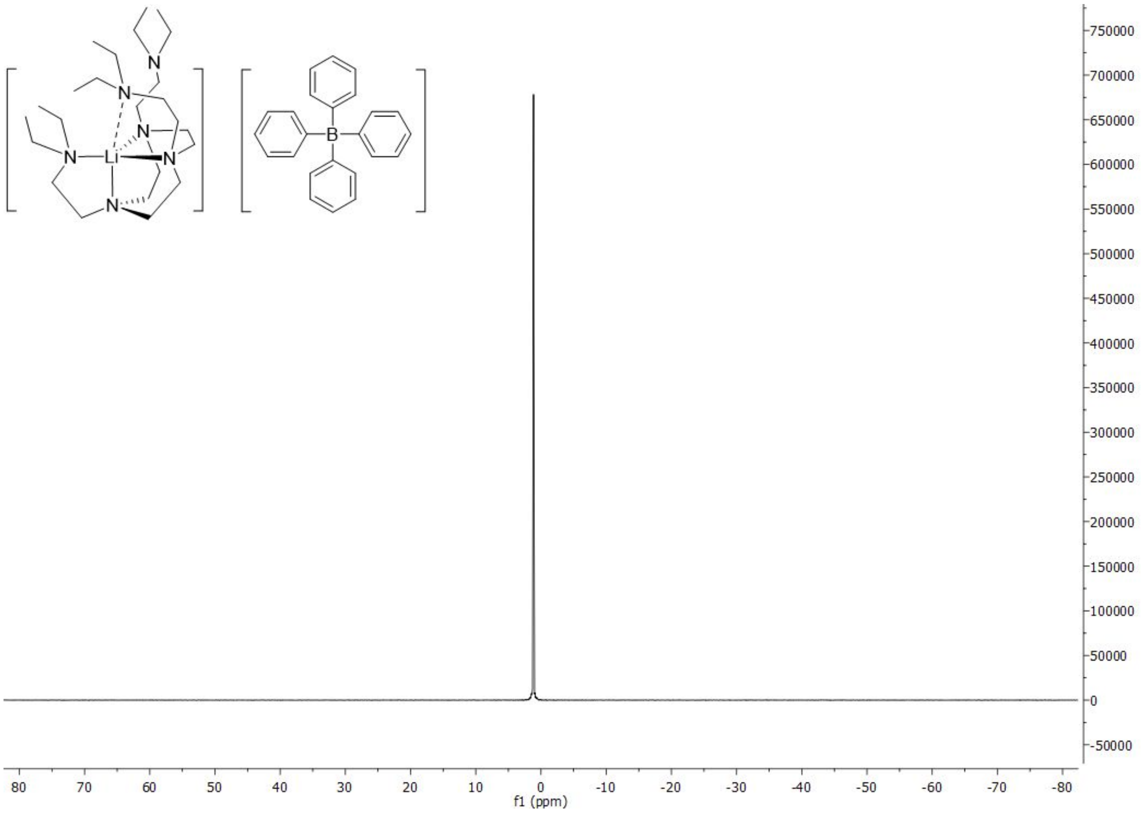


Figure S13. $^{7}$Li NMR Spectrum of complex **5** [Li($\kappa^3$-*N,N',N''*-DETAN)][$BPh_4$].

## Synthesis of [Na(I)($\kappa^3$-*N,N',N''*-$Me_6$Tren)] (**6**)

At room temperature, a $Me_6$Tren-THF solution (0.461 g of $Me_6$Tren, 2 mmol, in 6 mL of THF) was added to solid NaI (0.300 g, 2 mmol) with vigorous stirring. The resulting white suspension was heated to 50 ˚C for 3 days to form a white suspension as **6** is only partially soluble in THF at 50 ˚C. The suspension was refluxed and filtered when it is warm. The colourless filtrate was allowed to cool to room temperature, afford colourless crystals. The mother liquor was removed, and the crystals were washed with hexane (2 mL x 3) and dried *in vacuo*, to afford **6** as a white crystalline solid (0.332 g, 44% yield). Single crystals suitable for SXCRD were obtained by slowly cooling saturated refluxing solution of **2** in THF.

$^{1}$H NMR (300 MHz, $CDCl_3$, 25 °C): δ (ppm) 2.44 (m, 6H, N*$CH_2$*), 2.35 (m, 6H, N*$CH_2$*), 2.28 (s, 18H, N*$CH_3$*$)_2$).

$^{13}$C{$^{1}$H} NMR (75 MHz, $CDCl_3$, 25 °C): δ (ppm) 56.7 (N*C*$H_2CH_2$N), 50.7 (N$CH_2$*C*$H_2$N), 45.3 (N*C*$H_3)_2$).

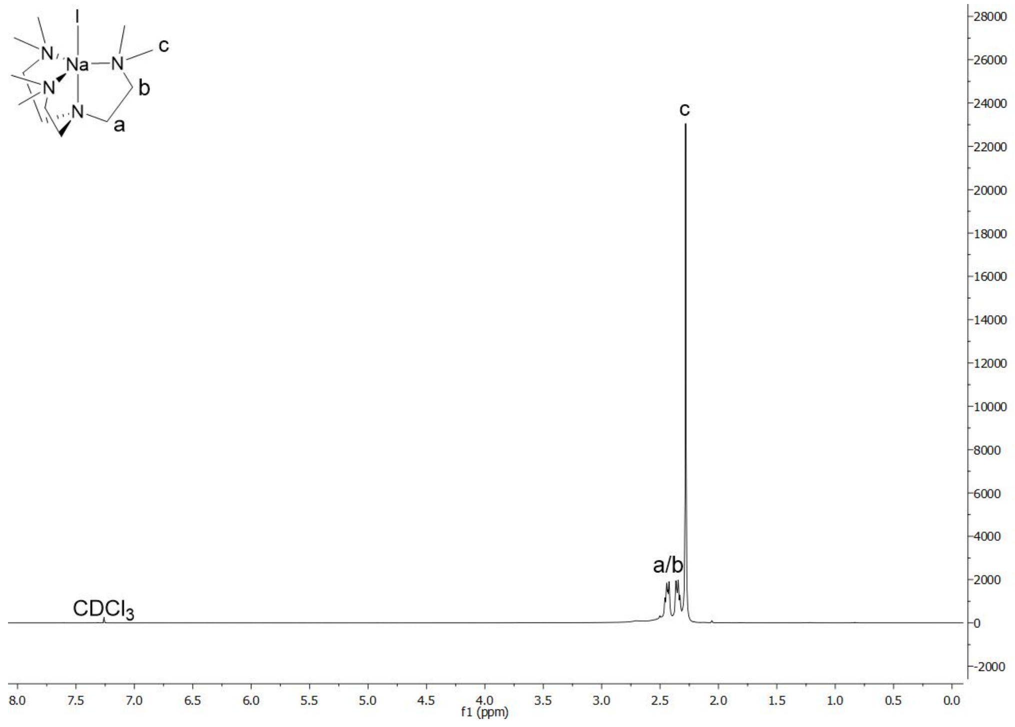


Figure S14. $^{1}$H NMR Spectrum of complex **6** [Na(I)(*κ*[3]-*N,N'N''*-$Me_6$Tren)].

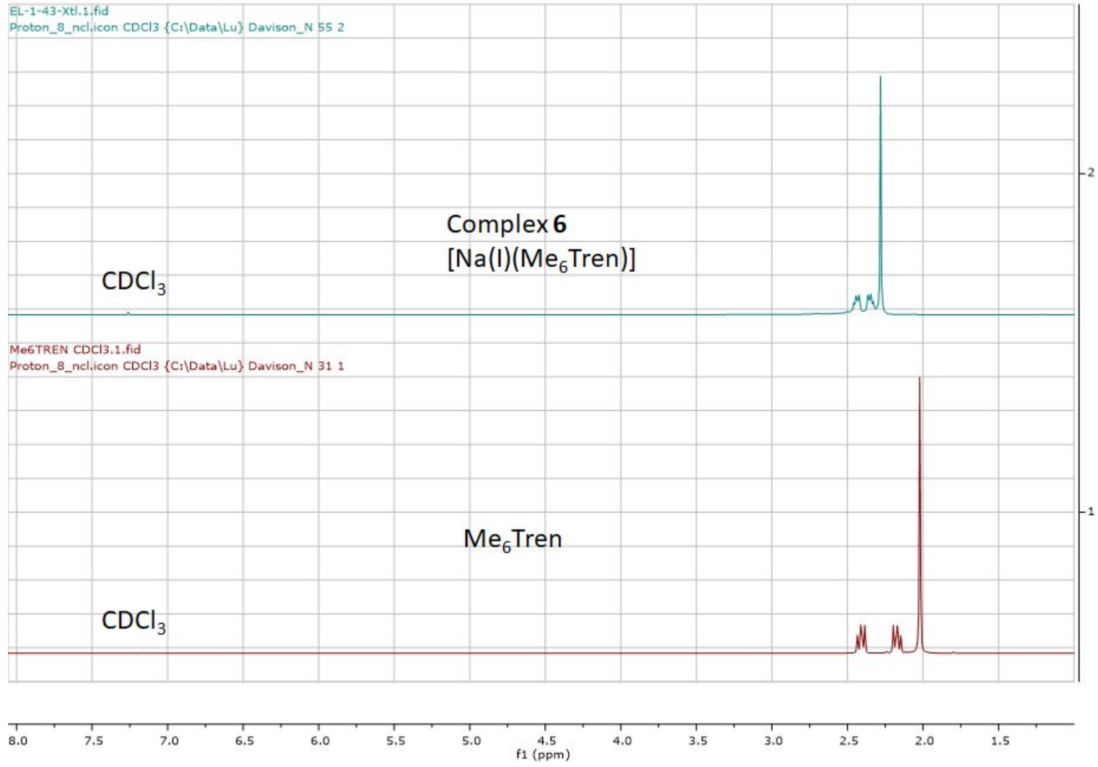

Figure S15. $^1$H NMR Spectra comparison between **6** and $Me_6$Tren in $CDCl_3$ at room temperature.

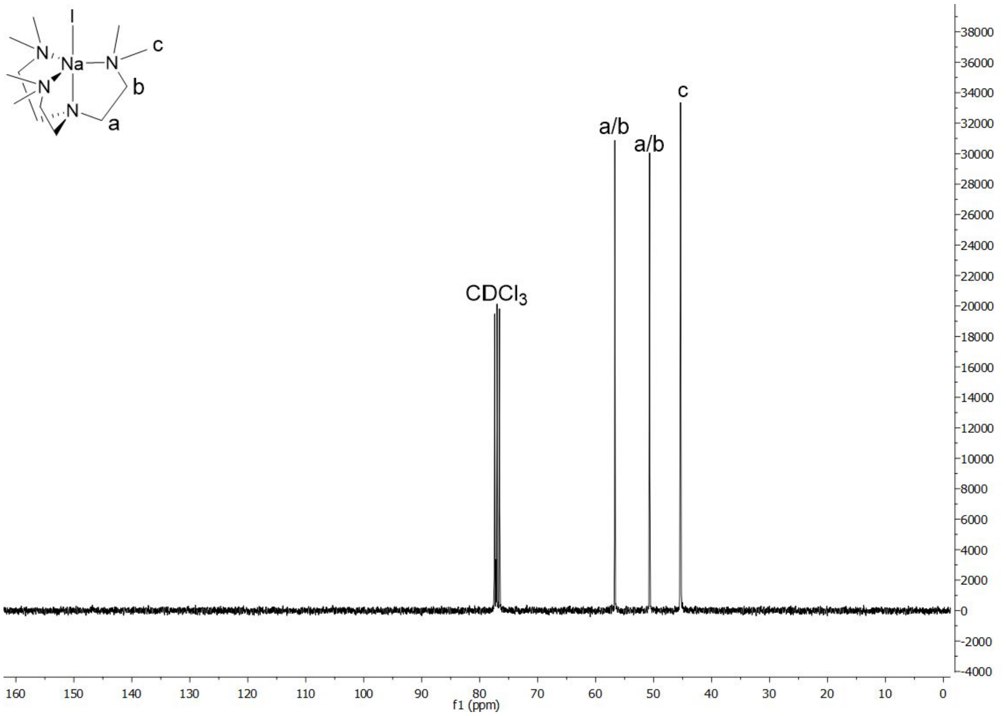


Figure S16. $^{13}C\{^1H\}$ NMR Spectrum of complex **6** [Na(I)(*κ³-N,N'N''*-Me⁶Tren)]

## Synthesis of [Na(I)(DETAN)] (**7**)

DETAN (1.280 g, 3 mmol) and NaI (0.450 g, 3 mmol) were combined in a 100 ml Schlenk flask. At room temperature, 20 mL of THF was added to the mixture, and the resulting colourless solution was stirred at room temperature for 18 hours. All volatiles were removed *in vacuo* to afford a thick colourless oil, which was subsequently washed with hexane (10 ml) and dried *in vacuo* to afford **7** as a white solid (1.73 g, 76% yield).
$^1$H NMR (300 MHz, $CDCl_3$, 25 °C): δ (ppm) 2.72 (m, 6H, $NCH_2$), 2.61 – 2.46 (m, 30H, $NCH_2$), 0.96 (t, $^3J_{HH}$ = 7.2 Hz, 18H, $NCH_2CH_3$).
$^{13}C\{^1H\}$ NMR (75 MHz, $CDCl_3$, 25 °C): δ (ppm) 56.4, 52.2, 49.8 and 46.3 ($NCH_2$), 10.8 ($CH_2CH_3$).

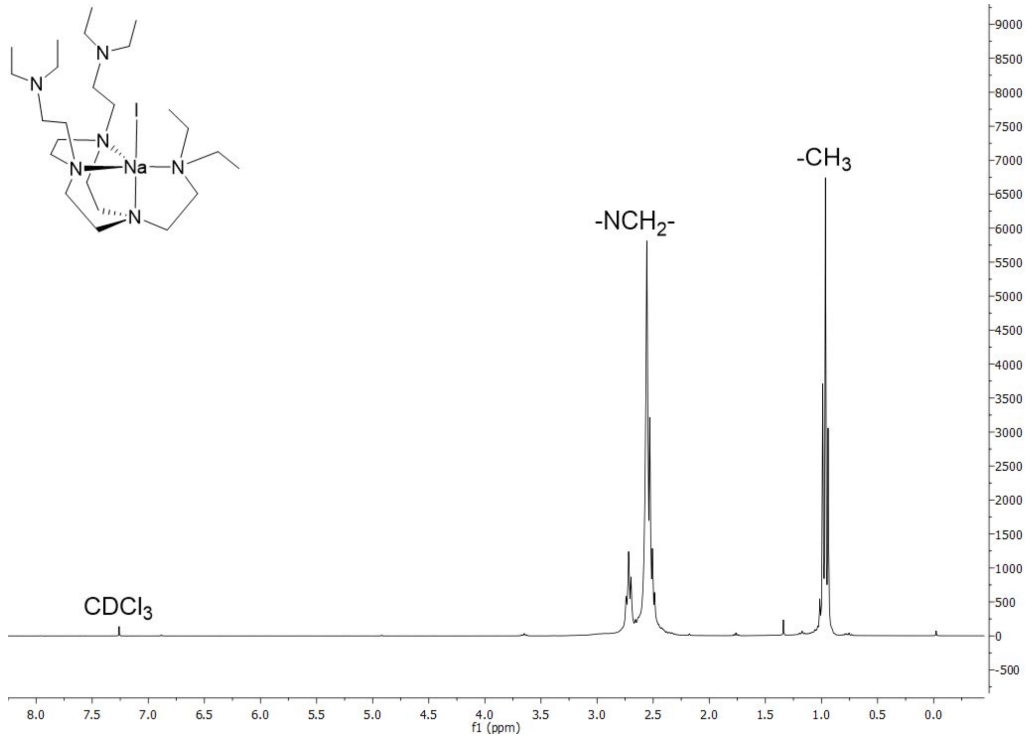


Figure S17. $^{1}$H NMR Spectrum of complex **7** [Na(I)(DETAN)].

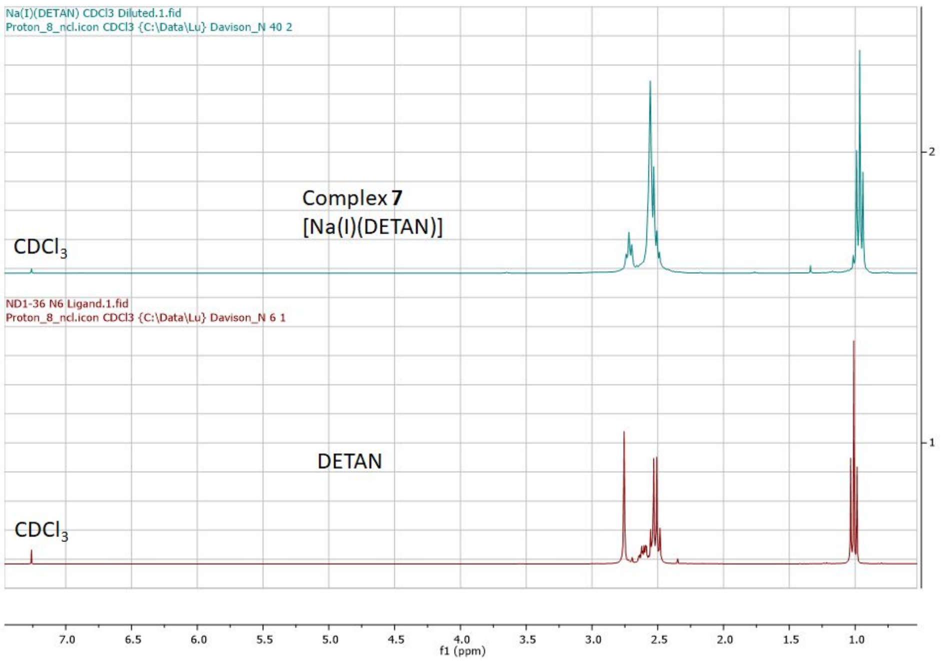


Figure S18. $^{1}$H NMR Spectra comparison between **7** and DETAN in $CDCl_3$ at room temperature.

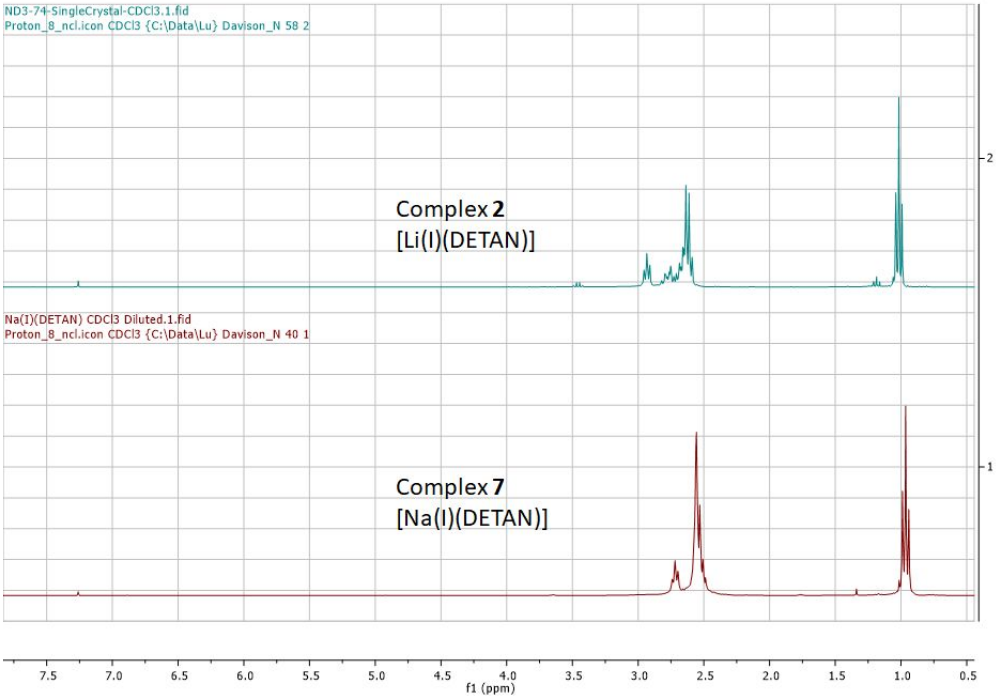


Figure S19. $^{1}H$ NMR Spectra comparison between **2** and **7** in $CDCl_3$ at room temperature.

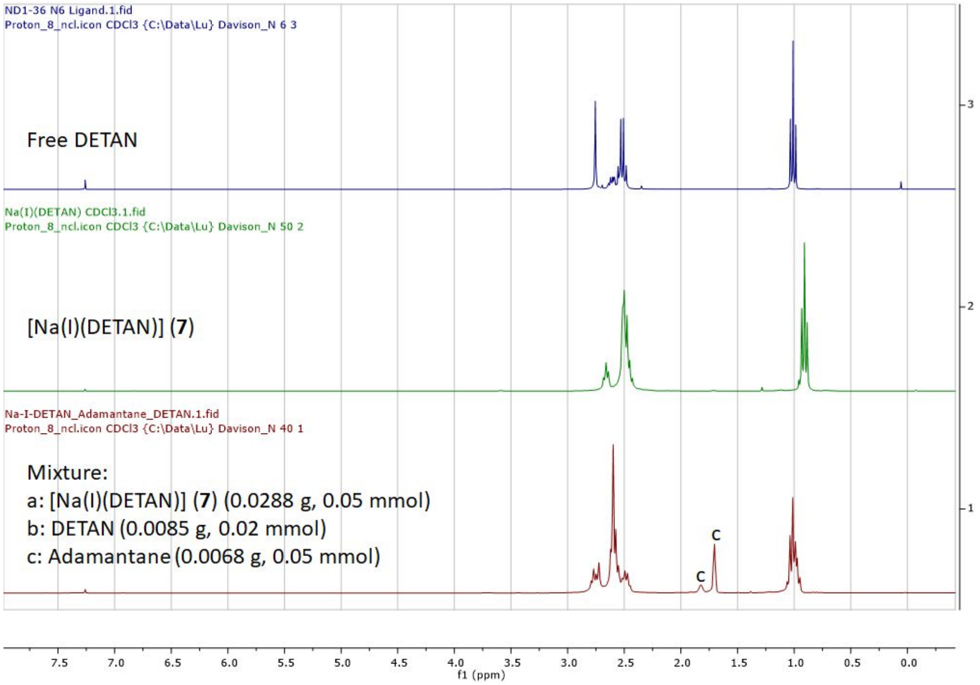


Fogure S20. $^{1}H$ NMR Spectra comparison between **7**, a mixture of 7, DETAN and adamantane (internal standard), and pure DETAN in $CDCl_3$ at room temperature.

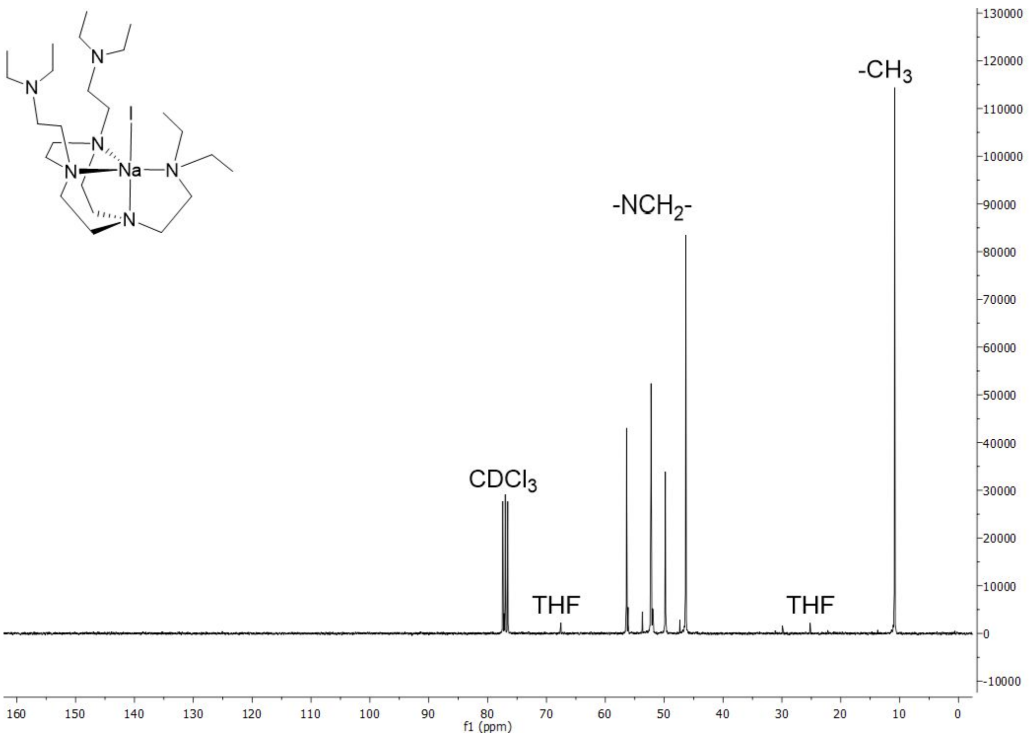


Figure S21. $^{13}C\{^{1}H\}$ NMR Spectrum of complex **7** [Na(I)(DETAN)].

Synthesis of [Na(DETAN)][$BPh_4$] (**8**)

At room temperature, a DETAN (0.427 g, 1 mmol) solution in $Et_2O$ (1 ml) was added to a $NaBPh_4$ (0.442 g, 1 mmol) suspension in $Et_2O$ (1 ml) in one-portion, followed by additional 4 mL of THF. The resulting colourless solution was stirred at room temperature for 18 hours. All volatiles were removed *in vacuo* to afford a white solid, which was washed with hexane (10 ml) and dried *in vacuo*, to afford **8** as a white solid (0.769 g, 82% yield). Single crystals suitable for SCXRD were obtained from a $Et_2O$/THF (1 mL/0.3 mL) solution of 0.158 g of **8** after standing at -35 ˚C for 24 hours.

$^{1}H$ NMR (300 MHz, $CDCl_3$, 25 °C): δ (ppm) 7.46 (m, 8H, Ar-*H*s), 7.10 (t, $^{3}J_{HH}$ = 7.3 Hz, 8H, Ar-*H*s), 6.95 (m, 4H, Ar-*H*s), 2.66 – 2.42 (m, 30H, $NCH_2$), 2.35 – 2.22 (m, 6H, $NCH_2$), 1.02 (t, $^{3}J_{HH}$ = 7.1 Hz, 18H, $NCH_2CH_3$).

$^{13}C\{^{1}H\}$ NMR (75 MHz, $CDCl_3$, 25 °C): δ (ppm) 164.1 (q, $^{1}J_{BC}$ = 46.7 Hz, Ar-$C_{ipso}$), 136.2, 125.3 and 121.4 (Ar-*C*s), 56.7, 53.4, 50.5 and 45.7 ($NCH_2$), 9.6 ($CH_2CH_3$).

$^{11}B$ NMR (96 MHz, $CDCl_3$, 25 °C): δ (ppm) -6.48.

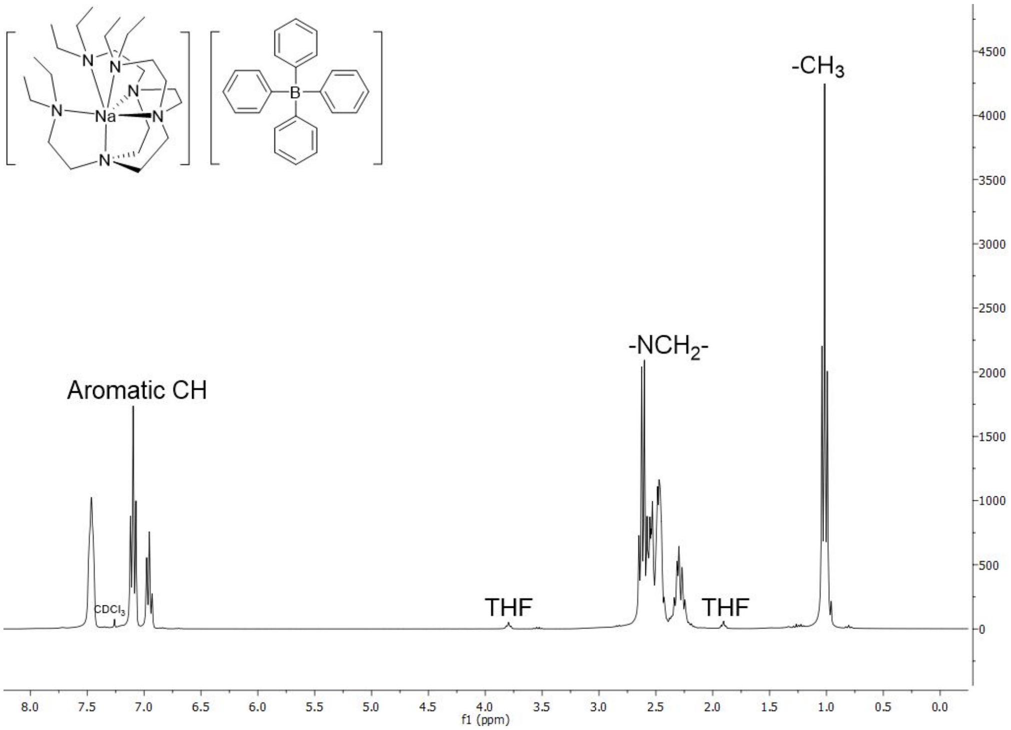


Figure S22. $^{1}$H NMR Spectrum of complex [Na(DETAN)][$BPh_4$] (**8**).

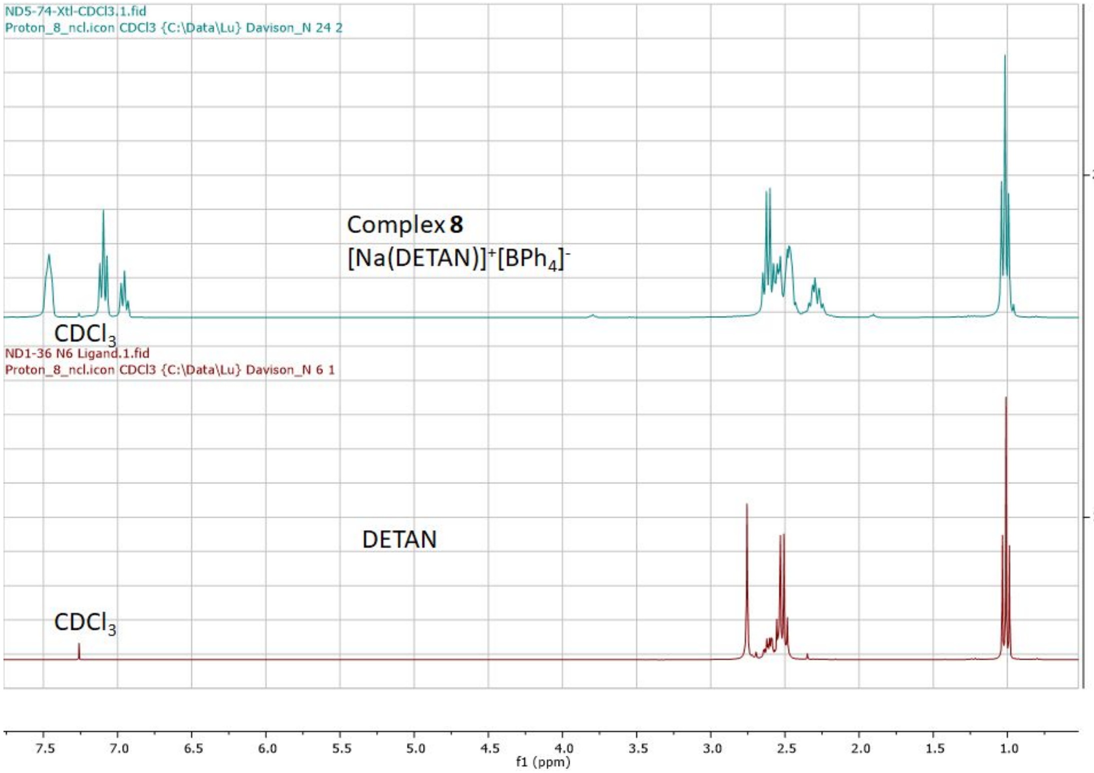


Figure S23. $^{1}$H NMR Spectra comparison between **8** and DETAN in $CDCl_3$ at room temperature.

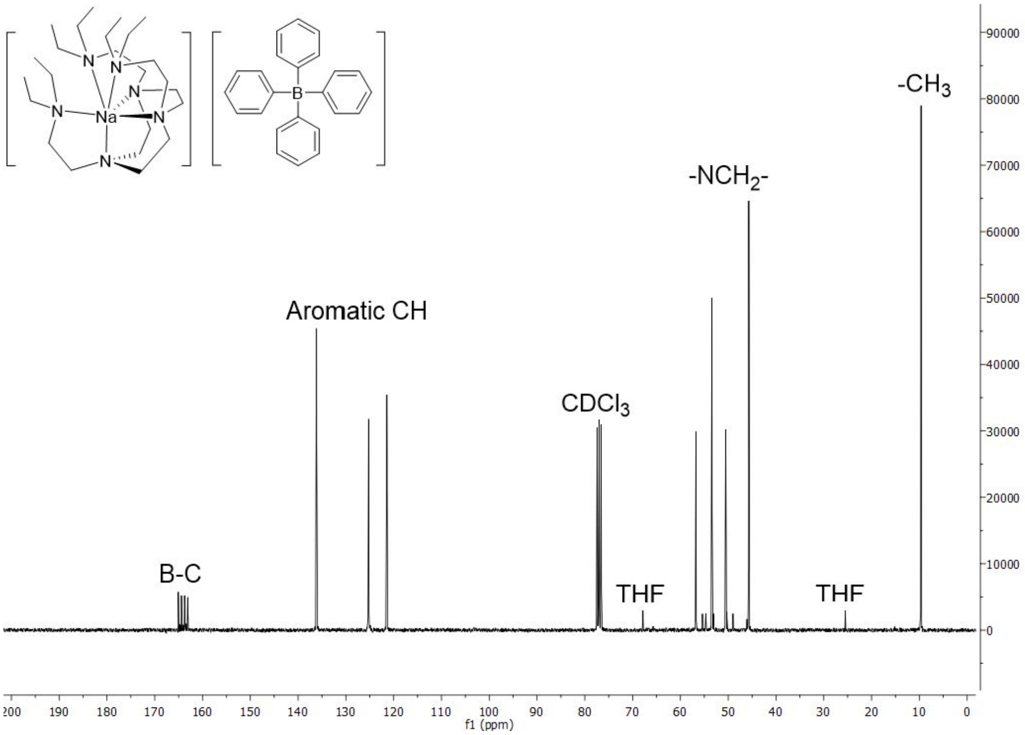


Figure S24. $^{13}C\{^{1}H\}$ NMR Spectrum of complex [Na(DETAN)][$BPh_4$] (**8**).

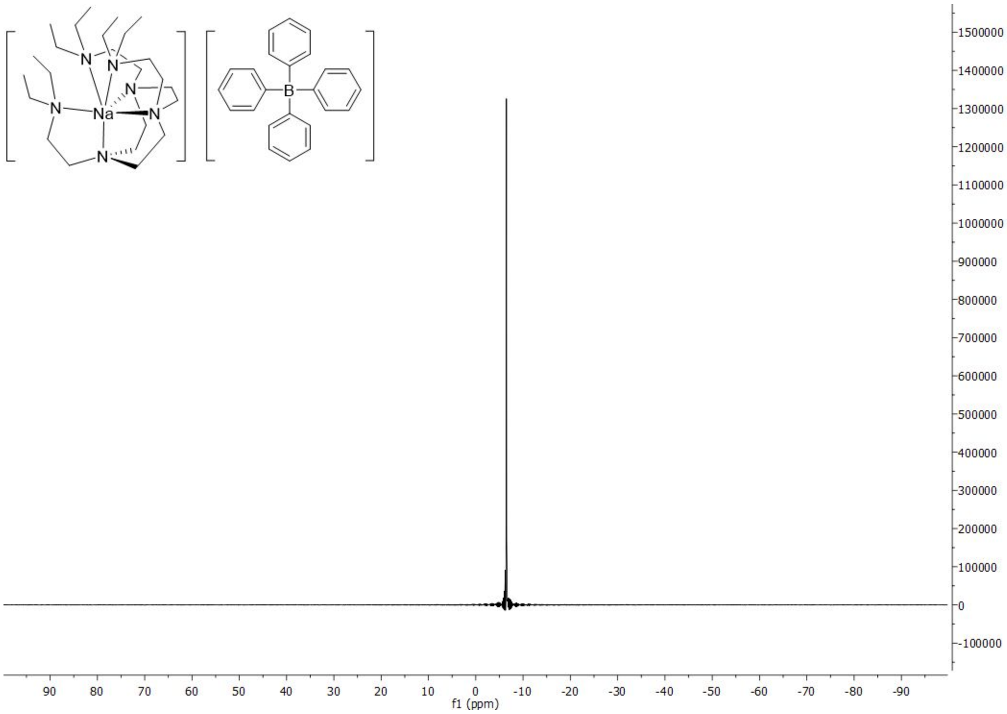


Figure S25. $^{11}B$ NMR Spectrum of complex [Na(DETAN)][$BPh_4$] (**8**).

## Reactions between KI/$KBPh_4$/RbI/CsI and one equivalent of DETAN/$Me_6$Tren

The DETAN/$Me_6$Tren (0.025 mmol) and KI/$KBPh_4$/RbI/CsI (0.025 mmol) mixtures were heated at 50 °C in 0.5 mL of $d_8$-THF (J. Young tap NMR tube) for two days.

Alternatively, the mixtures of $KBPh_4$ (1.0 mmol) and $Me_6$Tren/DETAN (1.0 mmol) were heated at 100 °C in 10 mL of toluene in sealed J. Young tap ampoule for one day.

The $^1$H NMR spectra indicate no reactions took place for all the cases (only signals for the DETAN/$Me^6$Tren ligands).

## DOSY NMR Studies of complexes **2** and **7**

DOSY NMR experiments were performed on a Bruker 700 Avance III HD NMR spectrometer using a TCI cryoprobe with a maximum gradient strength of 60 G cm-1. Samples were prepared using 15 mM of complexes **2** or **7**, and an equimolar ratio of adamantane in $CD_2Cl_2$. The method of using DOSY to estimate MW as outlined in the Neufeld and Stalke work[2]. The Bruker dstebpgp3s pulse sequence was used with a diffusion time $\Delta$ of 0.1 s and magnetic field pulse gradient ($\delta/2$) of 800 μs. Diffusion coefficients were calculated with the T1/T2 software in Topspin and the DOSY plot was generated using Bruker's Dynamic centre.

We employed a relaxation delay time ($d_1$) of 5 seconds and acquired 16 spectra for each DOSY experiment leading to a total acquisition time of 35 minutes. The DOSY NMR spectra of the mixtures, **2 +** adamantane (ADAM) and **7** + ADAM, are displayed in Figure S25 and S26, respectively.

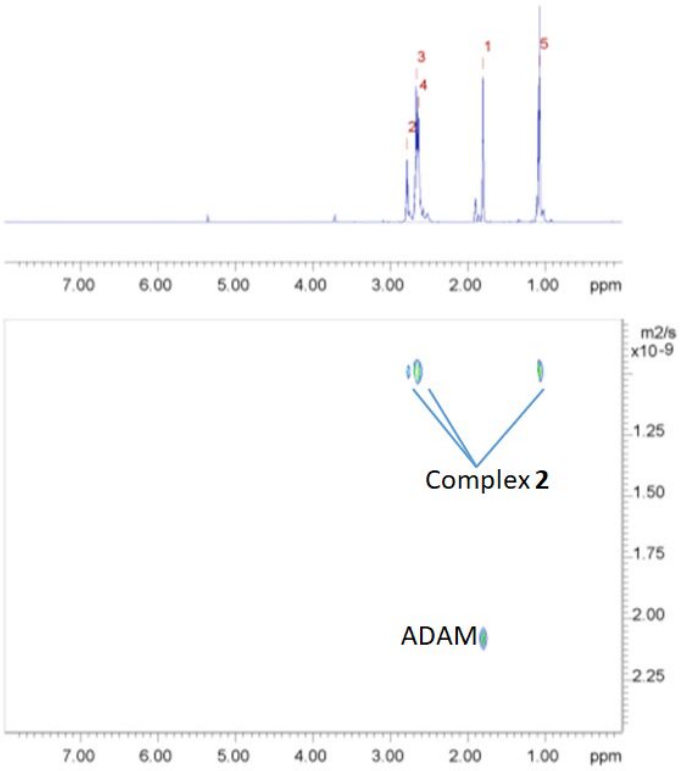


Figure S26. The $^{1}$H DOSY NMR spectrum of complex **2** and the internal standard adamantane (ADAM).

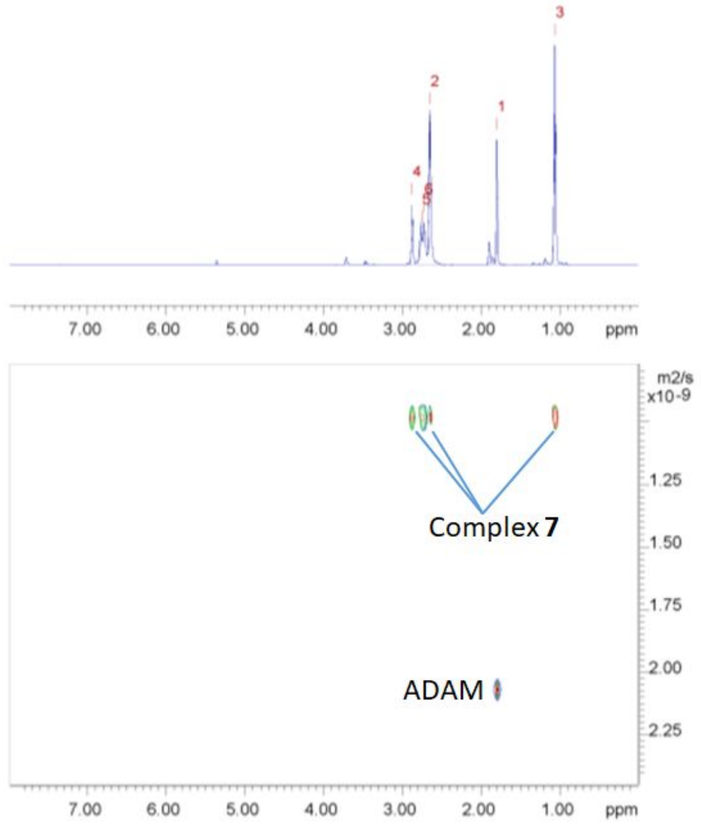


Figure S27. The $^1$H DOSY NMR spectrum of complex **7** and the internal standard adamantane (ADAM).

| Complex | **1** | **2** | **5** | **6** | **8** |
|---|---|---|---|---|---|
| Empirical formula | $C_{12}H_{30}ILiN_4$ | $C_{24}H_{54}ILiN_6$ | $C_{48}H_{74}BLiN_6$ | $C_{12}H_{30}IN_4Na$ | $C_{48}H_{74}BN_6Na$ |
| Formula weight | 364.24 | 560.57 | 752.88 | 380.29 | 768.93 |
| Temperature/K | 150(2) | 150(2) | 150(2) | 150(2) | 150(2) |
| Crystal system | hexagonal | monoclinic | triclinic | hexagonal | monoclinic |
| Space group | $P6_3$ | $P2_1/n$ | P-1 | $P6_3$ | $P2_1/n$ |
| a/Å | 9.9201(3) | 8.92410(10) | 11.5876(4) | 10.2321(2) | 12.2838(5) |
| b/Å | 9.9201(3) | 15.14560(10) | 12.6450(5) | 10.2321(2) | 19.1933(8) |
| c/Å | 10.3869(3) | 21.9219(2) | 17.9025(7) | 10.2301(2) | 19.7594(7) |
| α/° | 90 | 90 | 83.823(3) | 90 | 90 |
| β/° | 90 | 98.1960(10) | 75.482(3) | 90 | 100.859(4) |
| γ/° | 120 | 90 | 62.747(4) | 120 | 90 |
| Volume/Å$^3$ | 885.22(5) | 2932.72(5) | 2257.44(17) | 927.56(4) | 4575.2(3) |
| Z | 2 | 4 | 2 | 2 | 4 |
| $\rho_{calc}$g/cm$^3$ | 1.367 | 1.27 | 1.108 | 1.362 | 1.116 |
| μ/mm$^{-1}$ | 14.12 | 8.72 | 0.483 | 13.72 | 0.576 |
| F(000) | 372 | 1184 | 824 | 388 | 1680 |
| Crystal size/mm$^3$ | 0.39 × 0.095 × 0.076 | 0.4 × 0.1 × 0.06 | 0.3 × 0.25 × 0.14 | 0.35 × 0.07 × 0.05 | 0.29 × 0.23 × 0.11 |
| Radiation | Cu Kα (λ = 1.54184) | Cu Kα (λ = 1.54184) | Cu Kα (λ = 1.54184) | Cu Kα (λ = 1.54184) | Cu Kα (λ = 1.54184) |
| 2Θ range for data collection/° | 10.296 to 132.964 | 7.118 to 133.164 | 7.866 to 133.234 | 9.982 to 132.916 | 3.8810 to 65.0380 |
| Index ranges | -11 ≤ h ≤ 11, -11 ≤ k ≤ 11, -12 ≤ l ≤ 12 | -8 ≤ h ≤ 10, -18 ≤ k ≤ 18, -26 ≤ l ≤ 26 | -9 ≤ h ≤ 13, -14 ≤ k ≤ 15, -21 ≤ l ≤ 21 | -12 ≤ h ≤ 12, -12 ≤ k ≤ 12, -11 ≤ l ≤ 12 | -14 ≤ h ≤ 14, -19 ≤ k ≤ 22, -23 ≤ l ≤ 23 |
| Reflections collected | 8378 | 42012 | 31835 | 12992 | 39384 |
| Independent reflections | 1053 [$R_{int}$ = 0.0603, $R_{sigma}$ = 0.0275] | 5169 [$R_{int}$ = 0.0652, $R_{sigma}$ = 0.0317] | 7914 [$R_{int}$ = 0.0474, $R_{sigma}$ = 0.0313] | 1080 [$R_{int}$ = 0.0592, $R_{sigma}$ = 0.0277] | 8091 [$R_{int}$ = 0.0789, $R_{sigma}$ = 0.0682] |
| Data/restraints/parameters | 1053/1/58 | 5169/0/295 | 7914/1282/714 | 1080/1/58 | 8019/1836/788 |
| Goodness-of-fit on F$^2$ | 1.073 | 1.026 | 1.02 | 1.12 | 1.043 |
| Final R indexes [I>=2σ (I)] | $R_1$ = 0.0275, $wR_2$ = 0.0678 | $R_1$ = 0.0365, $wR_2$ = 0.0908 | $R_1$ = 0.0697, $wR_2$ = 0.1969 | $R_1$ = 0.0428, $wR_2$ = 0.1109 | $R_1$ = 0.0682, $wR_2$ = 0.2143 |
| Final R indexes [all data] | $R_1$ = 0.0307, $wR_2$ = 0.0701 | $R_1$ = 0.0428, $wR_2$ = 0.0962 | $R_1$ = 0.0808, $wR_2$ = 0.2118 | $R_1$ = 0.0448, $wR_2$ = 0.1125 | $R_1$ = 0.0682, $wR_2$ = 0.2143 |
| Largest diff. peak/hole / e Å$^{-3}$ | 0.42/-0.53 | 1.17/-0.77 | 0.39/-0.36 | 0.82/-0.38 | 0.50/-0.36 |

## Computational Details

### *General*

Optimizations were carried out with the PBE[3], B3LYP[4], B3PW91[5] and PBE0[6] functionals along with relativistic effective core potential basis set SDD for iodine element and two types of basis sets (def2-TZVPP, 6-31G and 6-31+G) for other elements[7,8]. Dispersion correction in vacuum and in THF solution by using the integral equation formulation of polarizable continuum model (IEFPCM)[9] to take into account the solvent effect of THF within the Gaussian 09 package[10].

Table S1. calculated bond lengths (Å)

| | Calc. | | Expl. | |
|---|---|---|---|---|
| Å | Complex **1** | Complex **2** | Complex **1** | Complex **2** |
| Li(1)-N(2) | 2.26236 | 2.24259 | 2.222 | 2.179 |
| Li(1)-N(3) | 2.25748 | 2.15837 | 2.222 | 2.149 |
| Li(1)-N(4) | 2.25408 | 2.21668 | 2.222 | 2.18 |
| Li(1)-N(5) | 2.251 | 2.206 | 2.196 | 2.173 |
| Li(1)-I(6) | 2.966 | 3.166 | 2.979 | 3.11 |

Table S2. NBO charge on selected atoms in complexes-1 and -2.

| Q | Comp-1 | Comp-2 | ΔQ |
|---|---|---|---|
| Li(1) | 0.472 | 0.475 | 0.003 |
| N(2) | -0.544 | -0.563 | -0.019 |
| N(3) | -0.544 | -0.575 | -0.031 |
| N(4) | -0.544 | -0.565 | -0.021 |
| N(5) | -0.547 | -0.557 | -0.010 |
| I(6) | -0.721 | -0.728 | -0.007 |

Table S3 The calculated natural localized molecular orbitals (NLMOs) of Li and I in **1** and **2**.

| Species | Complex-1 | | Complex-2 | |
|---|---|---|---|---|
| | *Type* | NLMO | *Type* | NLMO |
| Li-I | 1 | 0.27%Li($sp^{2.23}$)+99.55%I($sp^{1.55}$) | 1 | 0.24%Li($sp^{1.34}$)+99.54%I($sp^{1.22}$) |
| | 2 | 3.46%Li($sp^{0.05}$)+96.19%I($sp^{0.64}$) | 2 | 3.18%Li($sp^{0.06}$)+96.14%I($sp^{0.87}$) |

Table S4 Energy decomposition analysis (EDA, in kcal/mol) for 1 and 2 according to the two fragments of $I^-$ and the $LiL^+$ ligand at B3LYP/6-31G/SDD levels.

| | 1 | 2 |
|---|---|---|
| Pauli Repulsion | 26.12 | 28.73 |
| Electrostatic Interaction | -92.73 | -88.18 |
| Orbital Interaction | -18.87 | -21.51 |
| Total Binding Energy | -85.48 | -80.96 |
| Covalency | 0.17 | 0.20 |

**References:**

1. N. Davison, E. Falbo, P. G. Waddell, T. J. Penfold and E. Lu, *Chem. Commun.,* 2021, **57**, 6205.
2. Neufeld, R., Stalke, D. *Chem. Sci.* 2015, **6**, 3354-3364.
3. J. P. Perdew, M. Emzerhof and K. Burke, *J. Chem. Phys.*, 1996, **105**, 9982.
4. A. D. Becke, *J. Chem. Phys.*, 1993, **98**, 5648.
5. C. T. Lee, W. T. Yang, R. G. Parr, *Phys. Rev. B*, 1988, **37**, 785.
6. A.D. Becke, *J.Chem.Phys.*,1993, **98**, 5648.
7. J. D. Dill and J. A. Pople, *J. Chem. Phys.*, 1975, **62**, 2921.
8. F. Weigend and R. Ahlrichs, *Phys. Chem. Chem. Phys.*, 2005, **7**, 3297.
9. Frisch, M. J.; Trucks, G. W.; Schlegel, H. B.; Scuseria, G. E.; Robb, M. A.; Cheeseman, J. R.; Scalmani, G.; Barone, V.; Mennucci, B.; Petersson, G. A.; Nakatsuji, H.; Caricato, M.; Li, X.; Hratchian, H. P.; Izmaylov, A. F.; Bloino, J.; Zheng, G.; Sonnenberg, J. L.; Hada, M.; Ehara, M.; Toyota, K.; Fukuda, R.; Hasegawa, J.; Ishida, M.; Nakajima, T.; Honda, Y.; Kitao, O.; Nakai, H.; Vreven, T.; Montgomery Jr., J. A.; Peralta, J. E.; Ogliaro, F.; Bearpark, M.; Heyd, J. J.; Brothers, E.; Kudin, K. N.; Staroverov, V. N.; Keith, T.; Kobayashi, R.; Normand, J.; Raghavachari, K.; Rendell, A.; Burant, J. C.; Iyengar, S. S.; Tomasi, J.; Cossi, M.; Rega, N.; Millam, J. M.; Klene, M.; Knox, J. E.; Cross, J. B.; Bakken, V.; Adamo, C.; Jaramillo, J.; Gomperts, R.; Stratmann, R. E.; Yazyev, O.; Austin, A. J.; Cammi, R.; Pomelli, C.; Ochterski, J. W.; Martin, R. L.; Morokuma, K.; Zakrzewski, V. G.; Voth, G. A.; Salvador, P.; Dannenberg, J. J.; Dapprich, S.; Daniels, A. D.; Farkas, Ö.; Foresman, J. B.; Ortiz, J. V.; Cioslowski, J.; Fox, D. J., Gaussian 09, Revision C.01 Wallingford CT: Gaussian, Inc. 2010.
10. M. D. Gould, C. Taylor, S. K. Wolff, G. S. Chandler and D. Jayatilaka, *Theor. Chem. Acc.*, 2008, **119**, 275.
